# A Fully Phosphane‐Substituted Disilene

**DOI:** 10.1002/anie.201701867

**Published:** 2017-04-12

**Authors:** Keith Izod, Peter Evans, Paul G. Waddell

**Affiliations:** ^1^ Main Group Chemistry Laboratories School of Chemistry Newcastle University Newcastle upon Tyne NE1 7RU UK

**Keywords:** crystal structures, DFT calculations, multiple bonds, phosphorus, silicon

## Abstract

There is growing interest in compounds containing functionalized E=E multiple bonds (E=Si, Ge, Sn, Pb) because of their potential to exhibit novel physical and chemical properties. However, compounds containing multiple functionalizations are rare, with scarcity increasing with increasing degree of substitution. The first ditetrelene R_2_E=ER_2_ in which the E=E bond is substituted by four heteroatoms (other than Si) is described. The tetraphosphadisilene {(Mes)_2_P}_2_Si=Si{P(Mes)_2_}_2_ (**7**) is readily isolated from the reaction between SiBr_4_ and [(Mes)_2_P]Li, the latter of which acts as a sacrificial reducing agent. The structure of **7** is presented, while the bonding in, and stability of **7** were probed using DFT calculations.

Since Lappert's landmark report of the distannene {(Me_3_Si)_2_CH}_2_Sn=Sn{CH(SiMe_3_)_2_}_2_,^1^ numerous examples of compounds containing multiple bonds between p‐block elements have been reported.^2^ Nevertheless, there is continuing interest in these compounds and recent attention has turned to functionalized species, which provide fascinating opportunities for tuning the physical and chemical properties of the E=E bond.^3^, ^4^, ^5^, ^6^, ^7^, ^8^, ^9^, ^10^, ^11^, ^12^

Typically, ditetrelenes (the heavier group 14 analogues of alkenes, R_2_E=ER_2_) require sterically demanding substituents to kinetically protect the E=E double bond.^1^, ^2^ These substituents are usually drawn from bulky aryl, silyl, or occasionally alkyl groups. In contrast, ditetrelenes substituted by heteroatoms from groups 15–17 are rather scarce, since such heteroatoms usually favor the formation of the corresponding tetrylenes R_2_E.

Over the last few years several examples of disilenes and digermenes substituted by either one or two group 15–17 atoms have been reported. For example, Scheschkewitz and co‐workers demonstrated that treatment of (Trip)_2_Si=Si(Trip)(Li) with R_2_PCl or iodine gives (Trip)_2_Si=Si(Trip)(PR_2_) (**A**, Figure 1) or (Trip)_2_Si=Si(Trip)(I) (**B**), respectively (R=Ph, *i*Pr, Cy, *t*Bu, NR′_2_ (R′=Me, Et, *i*Pr); Trip= 2,4,6‐*i*Pr_3_C_6_H_2_),^4^ while Sekiguchi and co‐workers showed that disilynes undergo 1,2‐addition with amines to give amino‐substituted disilenes [{(Me_3_Si)_2_CH}_2_ *i*PrSi](H)Si= Si(NR_2_)[Si*i*Pr{CH(SiMe_3_)_2_}_2_] (**C**; NR_2_=NEt_2_, N(CH_2_CH_2_)_2_, NPh_2_, and NH*t*Bu).^5^ In an alternative approach, Jutzi and co‐workers demonstrated that the disilene (Cp*){(Me_3_Si)_2_N}Si=Si{N(SiMe_3_)_2_}(Cp*) (**D**) may be formed (presumably via the transient silylene Cp*{(Me_3_Si)_2_N}Si:) by a reaction between [Cp*Si]^+^[B(C_6_F_5_)_4_]^−^ and Li[N(SiMe_3_)_2_].^6^ More recently, Roesky and co‐workers independently showed that this compound is accessible from the reaction of Cp*SiCl_2_H and K[N(SiMe_3_)_2_].^7^ Perhaps the most intriguing observation is that the cyclic diaminosilylene (CH_2_N*t*Bu)_2_Si: aggregates to an unusual amino‐substituted disilene **E** on standing at room temperature, rather than the corresponding tetraaminodisilene;^8^ in solution a dynamic equilibrium between the silylene and **E** is observed (closely related digermenes have been isolated by Weidenbruch and co‐workers).^9^ A dynamic equilibrium was also proposed for the highly sterically hindered dibromodisilene **F** and its bromosilylene analogue.^10^ Similarly, Müller, Kira, Apeloig, and co‐workers have proposed a dynamic equilibrium between the diaminosilylene (*i*Pr_2_N)_2_Si: and its disilene dimer, based on variable‐temperature UV/Vis spectroscopy; although this latter disilene has not been isolated and these studies suggest that the disilene itself is a minor component over the temperature range measured.^11^

**Figure 1:**
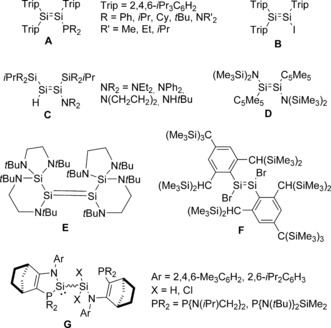
Disilenes substituted by group 14–17 atoms.

Very recently Bacereido, Kato, and co‐workers reported the dimerization of heteroleptic, intramolecularly base‐stabilized amino‐chlorosilylenes and amino‐hydrosilylenes by insertion of one silylene fragment into the Si−X bond of another to give compounds **G**.^13^ To date, there have been no reports of disilenes substituted by three or more group 15–17 atoms, which are isolable in the solid state.

Since the tendency for heteroatom‐substituted tetrylenes to dimerize to the corresponding ditetrelenes is a function of the electronegativity and π‐donor capacity of the heteroatom, diaminotetrylenes (R_2_N)_2_E (E=Si, Ge, Sn, Pb) typically show limited tendency towards dimerization. Phosphorus is significantly less electronegative than nitrogen and, while calculations suggest that P and N have similar inherent π‐donor capabilities,^14^ the large barrier to inversion at phosphorus prevents routine adoption of the planar geometry necessary for efficient pπ–pπ interactions. Thus, diphosphatetrylenes (R_2_P)_2_E represent an interesting class of compounds in which the tetrel center may not benefit from significant stabilization from the σ‐withdrawing/π‐donating effects of the phosphorus atoms. It is perhaps unsurprising then that few diphosphatetrylenes have been reported and that, until recently, none of these compounds exhibited P–E pπ–pπ interactions.^15^

Recently, we reported the first examples of diphosphagermylenes and ‐stannylenes (**H**) in which phosphorus adopts a planar geometry, resulting in efficient P–E pπ–pπ interactions (Figure 2).^16^ We now report our attempts to extend this method to the synthesis of a diphosphasilylene and the consequent isolation of a unique disilene substituted by four phosphorus atoms.

**Figure 2:**
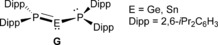
Diphosphagermylenes and ‐stannylenes.

Following on from our earlier report,^16^ we initially attempted the synthesis of the crowded precursor {(Dipp)_2_P}_2_SiCl_2_ (**1**), aiming to reduce this to the corresponding diphosphasilylene. However, while we were able to isolate a few crystals of **1** (Supporting Information), the difficulty of isolating this compound cleanly in acceptable yield prevented further exploitation. Consequently, we sought a less sterically demanding substituent at phosphorus and so synthesized the dichlorosilane {(Mes)_2_P}_2_SiCl_2_ (**2**), which was isolated in good yield and purity from the reaction between SiCl_4_ and 2 equivalents of [(Mes)_2_P]Li (Mes=2,4,6‐Me_3_C_6_H_2_), as a colorless solid.

However, treatment of **2** with 2 equivalents of KC_8_ did not lead to the corresponding diphosphasilylene {(Mes)_2_P}_2_Si: (**3**), but instead gave a mixture of the potassium phosphanide [(Mes)_2_P]K, along with a small amount of the diphosphane (Mes)_2_P−P(Mes)_2_ and a very small number of red crystals, which were shown by X‐ray crystallography to be the triphosphasilanate complex {(Mes)_2_P}_3_SiK(THF)_3_ (**4**) (Supporting Information). The formation of **4** during this reaction clearly indicates that the Si^IV^ center is reduced to Si^II^ in this process. However, given that **4** is effectively an adduct between **3** and [(Mes)_2_P]K, its formation suggests in situ reduction of **3** (or the corresponding disilene, see proceeding text) by the KC_8_ to give [(Mes)_2_P]K and an unidentified silicon‐containing species (Scheme 1). Attempts to circumvent this over‐reduction by using lithium as a milder reducing agent gave the triphosphasilanate complex {(Mes)_2_P}_3_SiLi(THF) (**5**.THF; identified by multielement NMR spectroscopy), whereas magnesium was found not to react with **2**. We have not been able to synthesize **4** by a more systematic route, but **5** may be isolated in good yield and purity (see proceeding text).

**Scheme 1:**
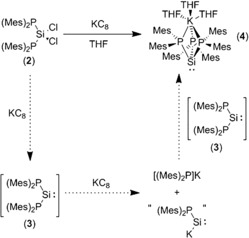
Reactivity of **2** with KC_8_ to form complex **4**.

It has been determined that the dehydrochlorination of chlorosilanes by strong bases is an effective route to Si^II^ species.^7^, ^17^ Therefore, as an alternative strategy we prepared the chlorosilane {(Mes)_2_P}_2_SiClH (**6**). However, reactions between **6** and a variety of strong non‐nucleophilic bases (such as LiN(SiMe_3_)_2_ or LiN(CMe_2_CH_2_)_2_CH_2_) gave complex mixtures of products, as judged by ^31^P{^1^H} NMR spectroscopy, from which we were not able to isolate any silicon‐containing species.

Since the over‐reduction of **2** by alkali metals is in direct competition with the formation of the diphosphasilylene **3**, we sought to prepare the dibromosilane {(Mes)_2_P}_2_SiBr_2_ in the expectation that reduction of this species to a diphosphasilylene would be more competitive with the over‐reduction (P−Si cleavage) process. To our surprise, we found that the reaction between SiBr_4_ and 2 equiv of [(Mes)_2_P]Li in diethyl ether gave a dark blue solution, which slowly decolorized and deposited a small amount of dark‐purple crystals; these were shown by X‐ray crystallography to be the tetraphosphadisilene {(Mes)_2_P}_2_Si=Si{P(Mes)_2_}_2_ (**7**) (see proceeding text).

This clearly indicated that the lithium phosphanide was itself acting as a reducing agent and so we adjusted the stoichiometry accordingly.^18^ Thus, the reaction between SiBr_4_ and four equivalents of [(Mes)_2_P]Li in diethyl ether yields a similar dark‐blue solution. Removal of the solvent and extraction of the residue into light petroleum gave a brown solution after removal of the LiBr side‐product (Scheme 2). A ^31^P{^1^H} NMR spectrum of this crude solution exhibits a singlet at −31.4 ppm that is due to (Mes)_2_P−P(Mes)_2_ and a broad singlet at −41.3 ppm, which we tentatively assign to the silylene **3** (although we have not yet been able to isolate this species), along with a 1:1:1:1 quartet at −62.5 ppm corresponding to **5**, and a singlet at −90.8 ppm that is due to an unknown species (approximate ratio of peaks 1.0:2.2:1.3:1.0). We were unable to locate a signal corresponding to **3** in the ^29^Si{^1^H} NMR spectrum of this crude solution; this may be due to extensive signal broadening for this species, as noted for the analogous diphosphastannylene **G** for which no ^119^Sn NMR signal could be found at room temperature either in the solid state or solution.^16^ On standing at room temperature for several days, this brown solution deposits dark‐purple crystals of **7** in reasonable yield; heating the solution under reflux accelerates deposition such that it is complete within 4 hours.

**Scheme 2:**
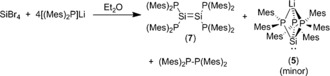
Synthesis of complexes **5** and **7** from SiBr_4_.

Compound **7** has limited solubility in common organic solvents, preventing characterization by solution‐state NMR spectroscopy. However, the solid‐state cross‐polarization magic angle spinning (CP‐MAS) ^31^P{^1^H} NMR spectrum of **7** consists of a pair of singlets at −55.9 and −77.9 ppm, consistent with the two distinct phosphorus environments observed by X‐ray crystallography (see proceeding text), while the solid‐state CP‐MAS ^29^Si{^1^H} NMR spectrum of **7** consists of a broad singlet at 111.7 ppm; ^31^P–^29^Si coupling is not resolved. The ^29^Si chemical shift of **7** is in the typical range for disilenes;^2^, ^3^ the observed ^31^P{^1^H} and ^29^Si{^1^H} chemical shifts correlate reasonably well with those obtained from DFT calculations (Supporting Information).

Single crystals suitable for X‐ray crystallography were grown from *n*‐hexane solutions of **3** that were left to stand at room temperature for several days. The structure of **7** is shown in Figure 3, along with selected bond lengths and angles. Compound **7** crystallizes as a discrete molecular species in which the silicon atoms are disordered over two positions with 92:8 occupancy, with a center of inversion midway along each of the two Si−Si vectors. The major disorder component has a strongly *trans*‐bent geometry (40.6° deviation of the SiP_2_ mean plane from the Si−Si vector). This contrasts with the near‐planar geometries adopted by most silicon‐substituted disilenes,^2^, ^3^ although a few carbon‐substituted disilenes do exhibit large *trans*‐bending angles.^19^ The *trans*‐bending angle in the major disorder component of **7** is similar to those observed in a few heteroatom‐substituted systems; for example in (Trip)_2_Si=Si(Trip){P(N*i*Pr_2_)_2_} the *trans*‐bending angle at the phosphorus‐substituted silicon center is 30.8°,^4^ while the *trans*‐bending angles in (Bbt)BrSi=SiBr(Bbt) are 32.4 and 39.8° (Bbt=2,6‐{(Me_3_Si)_2_CH}_2_‐4‐{(Me_3_Si)_3_C}C_6_H_2_).^10^

**Figure 3:**
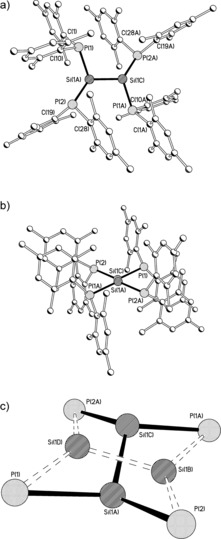
Molecular structure of the major disorder component of **7** viewed a) above and b) along the Si−Si vector (H atoms omitted for clarity). c) The core of **7**, showing the relationship of the two disorder components (minor component shown with dashed lines). Selected bond lengths [Å] and angles [°] for the major disorder component: Si(1A)−Si(1C) 2.1901(12), Si(1A)−P(1) 2.2666(8), Si(1A)−P(2) 2.2392(8), P(1)−C(1) 1.847(2), P(1)−C(10) 1.835(2), P(2)−C(19) 1.835(2), P(2)−C(28) 1.843(2); C(1)‐P(1)‐Si(1A) 116.34(8), C(10)‐P(1)‐Si(1A) 103.55(7), C(1)‐P(1)‐C(10) 105.73(10), C(19)‐P(2)‐Si(1A) 115.03(5), C(28)‐P(2)‐Si(1A) 112.76(7), C(28)‐P(2)‐C(19) 110.15(10), P(1)‐Si(1A)‐Si(1C) 108.19(4), P(2)‐Si(1A)‐Si(1C) 121.45(4), P(1)‐Si(1A)‐P(2) 113.52(3).

The phosphorus atoms adopt a pyramidal configuration (sum of angles at P(1) 325.62°, P(2) 337.94°), and the Si−P distances are 2.2666(8) and 2.2392(8) Å, which are comparable to Si−P distances reported for the few other compounds with a direct bond between P and Si^II^; for example, the Si−P distances in (Trip)_2_Si=Si(Trip)(PCy_2_) and {PhC(N*t*Bu)_2_}Si(P*i*Pr_2_) are 2.2367(12) and 2.307(8) Å, respectively.^4^, ^20^ The relatively short Si−P distances in **7** may suggest a degree of conjugation between the phosphorus lone pairs and the Si=Si bond. The plane of one aromatic ring on each phosphorus center lies parallel to the corresponding ring in the opposite Si(PR_2_)_2_ moiety with an interplane separation of 3.6 Å, consistent with an offset π–π interaction. This interaction may contribute to the overall stability of the disilene.

As a consequence of the restraints used in solving the crystal structure, any discussion of the minor disorder component of **7** must necessarily be more circumspect; however this disorder component appears to have a *trans*‐bending angle of 23.8°, while the Si(1B)−Si(1C) distance of 2.109(11) Å appears identical to that in the major disorder component. The minor disorder component was not observed in the solid‐state ^29^Si{^1^H} NMR spectrum of **7**, but a small peak is present at −67.4 ppm in the corresponding CP‐MAS ^31^P{^1^H} NMR spectrum, which we tentatively ascribe to this component.

Treatment of **7** with either lithium or KC_8_ yields the phosphanides [(Mes)_2_P]M (M=Li, K) as the sole identifiable phosphorus‐containing products. We also find that treatment of a slurry of **7** in THF with two equivalents of [(Mes)_2_P]Li(THF) cleanly yields the triphosphasilanate complex **5**.THF. The foregoing is consistent with our premise that the triphosphasilanate anions result from cleavage of a P−Si bond in **3** (or its dimer **7**), to generate [(Mes)_2_P]Li (or [(Mes)_2_P]K), followed by adduct formation with another molecule of **3** (or **7**) to give **4** or **5**.

To better understand the bonding in and stability of **7** we have undertaken a density functional theory (DFT) study. Since the X‐ray crystal structure of **7** contains two distinct disorder components, which differ chiefly in the degree of *trans*‐bending, we have modeled both of these molecules using the crystallographic coordinates as a starting point for the optimization; these are referred to hereafter as **7**_**maj**_ and **7**_**min**_ for the minimum energy geometries corresponding to the major and minor disorder components, respectively (Table 1). Additionally, we have located a minimum energy geometry for the alternative phosphanide‐bridged dimer {(Mes)_2_P}Si{μ‐P(Mes)_2_}_2_Si{P(Mes)_2_} (**7**_**alt**_).

**Table 1:** Calculated geometries for **7**_**maj**_, **7**_**min**_, **7**_**alt**_, **3**_**plan**_, and **3**_**pyr**_.

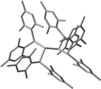	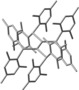	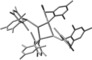
**7_maj_**	**7_min_**	**7_alt_**
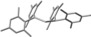		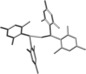
**3_plan_**		**3_pyr_**

The minimum energy geometry of **7**_**maj**_ is similar to the crystallographically determined structure, but exhibits a twist between the two SiP_2_ units (14.75° dihedral angle between the two normals of the SiP_2_ planes). The calculated *trans*‐bending angles of 38.42 and 40.04° are close to that determined crystallographically, but the calculated Si−Si bond distance of 2.2386 Å is somewhat longer than that observed in the solid state; this is likely a consequence of the twisted geometry of **7**_**maj**_, which would reduce the Si−Si π‐overlap. The calculated *trans*‐bending angle of **7**_**min**_ (6.22°) differs significantly from that in the solid‐state structure (23.8°), although the calculated Si−Si distance (2.182 Å) is close to that determined crystallographically (2.190(11) Å). The DFT calculations reveal that **7**_**min**_ and **7**_**maj**_ are almost isoenergetic, with the former just 9.0 kJ mol^−1^ less stable than the latter. In comparison, the alternative dimeric form **7**_**alt**_, containing a P_2_Si_2_ core, lies 82.7 kJ mol^−1^ higher in free energy than **7**_**maj**_.

Inspection of the molecular orbitals of both **7**_**maj**_ and **7**_**min**_ reveals that the HOMO and LUMO are essentially the Si=Si π and π* orbitals, although in both cases there is a significant component of these orbitals on the phosphorus atoms (Supporting Information). Natural Bond Orbital (NBO) analysis yields Wiberg Bond Indices (WBIs) for the Si=Si bonds in **7**_**maj**_ and **7**_**min**_ of 1.411 and 1.551, respectively, consistent with substantial double bond character. The WBIs for the Si−P bonds in **7**_**maj**_ and **7**_**min**_ range from 0.934 to 1.016, significantly greater than we calculate for a straightforward P−Si^II^ σ‐bond (see proceeding text), again suggesting some interaction between the phosphorus lone pairs and the Si=Si bond.

To explore the dimerization energy of the putative diphosphasilylene **3** to the tetraphosphadisilene **7** we have calculated the minimum energy geometries of the two extreme forms of the silylene. These are **3**_**plan**_, in which the two phosphorus centers approach planarity, and **3**_**pyr**_, in which both phosphorus centers adopt a pyramidal configuration (Table 1); all attempts to obtain a minimum energy geometry for a molecule possessing one planar and one pyramidal phosphorus center, as observed in the diphosphagermylenes and ‐stannylenes **H**, converged to **3**_**plan**_. For **3**_**plan**_ both phosphorus centers are close to planar (sum of angles at P=352.71 and 352.76°) and the Si−P distances (2.208 and 2.207 Å) are shorter than is typical for a Si−P single bond; however, the Si−P distances are longer than previously reported Si=P bonds in phosphasilenes such as (*t*Bu)(Trip)Si=P‐Si(*i*Pr)_3_ (Si=P 2.062(1), Si−P 2.255(1) Å),^21^ although we note that the latter involves Si^IV^ rather than Si^II^ and fully sp^2^‐hybridized and hence smaller phosphorus and silicon centers. The foregoing, along with Si−P WBIs of 1.221 and 1.222, suggest a significant Si−P π‐interaction in **3**_**plan**_, despite the slight variation from a planar geometry of the phosphorus centers. In contrast, the Si−P distances in **3**_**pyr**_ are both 2.337 Å and the WBIs for these bonds are both 0.804, consistent with Si−P single bonds.

Our calculations reveal that **3**_**plan**_ is more stable than **3**_**pyr**_ by 16.4 kJ mol^−1^. These calculations also show dimerization to the disilene **7**_**maj**_ is strongly favored; the difference in Gibbs free energy between the disilene **7**_**maj**_ and two equivalents of **3**_**plan**_ is +71.0 kJ mol^−1^. This is in marked contrast to recent calculations on the putative tetraamino‐substituted ditetrelenes {(Me_3_Si)_2_N}_2_E=E{N(SiMe_3_)_2_}_2_, which suggests that dissociation to the corresponding tetrylene monomers {(Me_3_Si)_2_N}_2_E: is strongly favored (E=Ge, Δ*G*=−69 kJ mol^−1^; E=Sn, Δ*G*=−75 kJ mol^−1^; E=Pb, Δ*G*=−45 kJ mol^−1^).^22^

In summary, we have shown that a unique tetraphosphadisilene is accessible by reduction of a simple Si^IV^ starting material, using sacrificial lithium phosphanide as the reducing agent. This is the first example of a structurally characterized ditetrelene substituted by more than two heteroatoms from groups 15–17.

## Conflict of interest

The authors declare no conflict of interest.

## Supporting information

As a service to our authors and readers, this journal provides supporting information supplied by the authors. Such materials are peer reviewed and may be re‐organized for online delivery, but are not copy‐edited or typeset. Technical support issues arising from supporting information (other than missing files) should be addressed to the authors.

Supplementary
