# Supplementary material for: A Fully Phosphane‐Substituted Disilene
Source: Angew Chem Int Ed Engl. 2017 Apr 12;56(20):5593–7. doi: 10.1002/anie.201701867 (PMC5450782; doi:10.1002/anie.201701867)
Supplement: Supplementary file 1 — Supplementary [file ANIE-56-5593-s001.pdf]

Angewandte Chemie
Eine Zeitschrift der Gesellschaft Deutscher Chemiker

Supporting Information

A Fully Phosphane-Substituted Disilene

Keith Izod, Peter Evans, and Paul G. Waddell*

anie_201701867_sm_miscellaneous_information.pdf

SUPPORTING INFORMATION

Contents:

S1-S6	Experimental details for [(Mes)$_2$P]Li(THF) and compounds **1**, **2**, **5**, **6**, and **7**.
S7	Table S1. Crystallographic details for **4**, and **7**.
S8	Figure S1. Molecular structure of **1**.
S10	Figure S2. Molecular structure of **4**.
S11-12	^{1}H, ^{13}C{^{1}H}, ^{7}Li and ^{31}P{^{1}H} NMR spectra of [(Mes)$_2$P]Li(THF).
S13-14	^{1}H, ^{13}C{^{1}H} ^{29}Si{^{1}H} and ^{31}P{^{1}H} NMR spectra of **2**.
S15-17	^{1}H, ^{13}C{^{1}H}, ^{7}Li, ^{29}Si{^{1}H} and ^{31}P{^{1}H} NMR spectra of **5.THF**.
S17-19	^{1}H, ^{13}C{^{1}H}, ^{29}Si and ^{31}P{^{1}H} NMR spectra of **6**.
S19-20	Solid-state CP-MAS ^{31}P{^{1}H} and ^{29}Si{^{1}H} NMR spectra of **7**.
S21	Details of DFT studies.
S21	Table S2. Calculated ^{31}P and ^{29}Si chemical shifts for **3$_{plan}$**, **3$_{pyr}$**, **7$_{maj}$**, **7$_{min}$**, and **7$_{alt}$**.
S21	Figure S3. HOMOs and LUMOs of **7$_{maj}$** and **7$_{min}$**.
S22-34	Final atomic coordinates and energies of **3$_{plan}$**, **3$_{pyr}$**, **7$_{maj}$**, **7$_{min}$**, and **7$_{alt}$**.
S34	References

Experimental Details

General: All manipulations were carried out using standard Schlenk and dry-box techniques under an atmosphere of dry nitrogen or argon. THF, diethyl ether, methylcyclohexane, *n*-hexane and light petroleum (b.p. 40-60 °C) were dried prior to use by distillation under nitrogen from sodium, potassium, or sodium/potassium alloy, as appropriate; hexamethyldisiloxane was dried over CaH_2. THF and hexamethyldisiloxane were stored over activated 4A molecular sieves; all other solvents were stored over a potassium film. Deuterated benzene was distilled from potassium under nitrogen and was deoxygenated by three freeze-pump-thaw cycles and was stored over activated 4A molecular sieves. $(Mes)_2PH$,[S1] and $(Dipp)_2PH$[S2] were prepared by previously published procedures; *n*-butyllithium was purchased from Aldrich as a 2.5 M solution in hexanes. All other compounds were used as supplied by the manufacturer.

^{1}H and $^{13}C\{^{1}H\}$ NMR spectra were recorded on a Bruker AvanceIII 500 spectrometer operating at 500.16 and 125.65 MHz, respectively, or a Bruker AvanceIII 300 spectrometer operating at 300.15 and 75.47 MHz, respectively; chemical shifts are quoted in ppm relative to tetramethylsilane. Solid-state $^{31}P\{^{1}H\}$ and $^{29}Si\{^{1}H\}$ NMR spectra were recorded at 161.99 and 79.51 MHz, respectively, using a Bruker AvanceIII 400 spectrometer and a 4 mm (rotor o.d.) magic-angle spinning probe. Spectra were obtained using cross-polarisation at ambient probe temperature (~25 °C) and at a sample spin-rate of either 6 or 10 kHz. Spectral referencing was with respect to an external sample of 85% H_3PO_4 (carried out by setting the signal from brushite to 1.0 ppm) or $SiMe_4$. Due to the air-sensitive nature of the compounds satisfactory elemental analyses could not be obtained.

Attempted synthesis of $\{(Dipp)_2P\}_2SiCl_2$ (1): To a solution of $Dipp_2PH$ (2.19 g, 6.17 mmol) in THF (25 mL) was added a solution of BnK (0.808 g, 6.17 mmol) in THF (20 mL). The resulting red solution was stirred for 30 min and added, dropwise, to a cold (-78 °C)

solution of $SiCl_4$ (0.35 mL, 3.05 mmol) in THF (15 mL). The resulting mixture was allowed to warm to room temperature to give a yellow solution with pale solids. A $^{31}P\{^1H\}$ NMR spectrum of this crude reaction mixture contained major peaks at -57 and -70 ppm in a 1:3.4 ratio, which were tentatively assigned as $(Dipp_2P)SiCl_3$ and $(Dipp_2P)_2SiCl_2$ respectively. Attempts to obtain **1** as a clean solid were unsuccessful, but a small number of single crystals suitable for X-ray crystallography were isolated from cold (-30 °C) methylcyclohexane.

Synthesis of [(Mes)$_2$P]Li(THF): This is a modification of a previously reported procedure.[S1] A solution of *n*BuLi in hexanes (2.3 M, 1.2 mL, 2.76 mmol) was added to a solution of Mes_2PH (0.76 g, 2.81 mmol) in THF (10 mL). The resulting dark red solution was stirred for 1 h then the solvent was removed *in vacuo*. The resulting sticky orange solid was dissolved in *n*-hexane (5 mL). Orange crystalline material was deposited on standing and the mixture was stored at -25 °C overnight. The supernatant was removed by filtration and the orange crystals were washed with cold (-10 °C) light petroleum (2 x 3 mL) and the residual solvent was removed under vacuum. Yield: 0.79 g, 81%. ^1H NMR [C_6D_6]: δ 1.05 (m, 4H, THF), 2.24 (s, 6H, *p*-Me), 2.41 (s, 12H, *o*-Me), 3.27 (m, 4H, THF), 6.92 (s, 4H, ArH). $^{13}C\{^1H\}$ NMR [C_6D_6]: δ 21.14 (*p*-Me), 25.18 (THF), 25.20 (*o*-Me), 68.68 (THF), 128.72 (ArH), 132.22, 140.54, 142.45 (Ar). $^7Li\{^1H\}$ NMR [C_6D_6]: δ 2.4 (br. s). $^{31}P\{^1H\}$ NMR [C_6D_6]: δ -90.4 (br. s).

Synthesis of (Mes$_2$P)$_2$SiCl$_2$ (2): To a solution of Mes_2PH (1.49 g, 5.51 mmol) in Et_2O (20 mL) was added a solution of *n*BuLi in hexanes (2.4 mmol, 5.5 mmol). The resulting orange solution was stirred for 1 h then added, dropwise, to a solution of $SiCl_4$ (0.3 mL, 2.6 mmol) in Et_2O (20 mL) at -78 °C to give a yellow solution with a pale precipitate that was allowed to warm to room temperature. The solvent was removed *in vacuo* to give a sticky pale yellow solid. The product was extracted into light petroleum (40 mL) and the resulting pale solids were removed by filtration. The solvent was removed *in vacuo* from the pale

yellow filtrate to give a pale yellow foam. The foam was dissolved in *n*-hexane (5 mL) and the product was precipitated as a white solid by the addition of hexamethyldisiloxane (10 mL). Further material was obtained by storage at -25 °C overnight. The supernatant was removed by filtration and the residual solvent was removed from the remaining white powder under vacuum. Yield: 1.33 g, 76%. ^{1}H NMR [C_6D_6]: δ 2.01 (s, 12H, o-Me), 2.59 (s, 24H, p-Me), 6.68 (s, 8H, ArH). ^{13}C{^{1}H} NMR [C_6D_6]: 20.85 (p-Me), 24.79 (t, J_{PC} = 8.1 Hz, o-Me), 128.90 (t, J_{PC} = 5.5 Hz, Ar), 130.11 (t, J_{PC} = 2.6 Hz, ArH), 138.65 (Ar), 144.16 (t, J_{PC} = 7.9 Hz, Ar). ^{29}Si{^{1}H}-INEPT NMR [C_6D_6]: 18.5 (t, J_{SiP} = 120 Hz). ^{31}P{^{1}H} NMR [C_6D_6]: δ -57.3.

Synthesis of ($Mes_2P)_2SiHCl$ (6): To a solution of Mes_2PH (1.70 g, 6.29 mmol) in Et_2O (30 mL) was added a solution of *n*BuLi in hexanes (2.7 mL, 6.21 mmol). The resulting orange solution was stirred for 1 h then added, dropwise, to a cold (-78 °C) solution of $SiHCl_3$ (0.3 mL, 2.97 mmol) in Et_2O (25 mL) to give a yellow solution with a pale precipitate that was allowed to warm to room temperature. The solvent was removed *in vacuo* to give a sticky pale yellow solid. The product was extracted into light petroleum (50 mL) and the resulting pale solids were removed by filtration. The solvent was removed *in vacuo* from the pale yellow filtrate to give a pale yellow foam. The foam was dissolved in *n*-hexane (5 mL) and the product was precipitated as a white solid by the addition of hexamethyldisiloxane (10 mL). Further material was obtained by storage at -25 °C overnight. The supernatant was removed by filtration and the residual solvent was removed under vacuum to give a white solid. Yield: 1.14 g, 71 %. ^{1}H NMR [C_6D_6]: δ 2.04 (s, 12H, *p*-Me), 2.48 (s, 24H, *o*-Me), 6.70 (s, 8H, ArH), 6.85 (t, J_{PH} = 20.0, 1H, SiH). ^{13}C{^{1}H} NMR [C_6D_6]: 20.90 (*p*-Me), 24.63 (t, J_{PC} = 8.2 Hz, *o*-Me), 129.78 (t, J_{PC} = 5.7 Hz, Ar), 130.03 (m, ArH), 138.10 (Ar), 143.64 (t, J_{PC} = 7.5 Hz, Ar). ^{29}Si-INEPT NMR [C_6D_6]: 2.4 (dt, J_{SiH} = 240 Hz, J_{SiP} = 80 Hz). ^{31}P{^{1}H} NMR [C_6D_6]: δ -75.8 (d, J_{PH} = 20.0 Hz).

Synthesis of $\{(Mes)_2P\}_2Si{=}Si\{P(Mes)_2\}_2$ (7): To a solution of Mes_2PH (1.09 g, 4.03 mmol) in Et_2O (20 mL) was added a solution of *n*BuLi in hexanes (2.3 M, 1.8 mL, 4.14 mmol). The resulting orange solution was stirred for 1 h and added, dropwise, over 30 min to a solution of $SiBr_4$ (2.0 mL of a 0.5 M solution in light petroleum, 1.0 mmol) in Et_2O (20 mL). The resulting dark blue solution gradually became brown while stirring at room temperature for 15 h. This crude reaction mixture contains 4 major species, as observed in the $^{31}P\{^1H\}$ NMR spectrum [-31.4 (s, Mes_2P-$PMes_2$), -41.3 (br. s), -62.5 (q, J_{PLi} = 44 Hz, $[(Mes_2P)_3Si]Li$) and -90.8 (br. s) in a 1.0:2.2:1.3:1.0 ratio, respectively]. The solvent was removed *in vacuo* from this solution and the product was extracted from the resulting sticky brown solid into *n*-hexane (25 mL). The resulting pale solids were removed by filtration and the filtrate was heated at reflux for 4 h, during which time **7** deposited as a dark purple microcrystalline solid. The supernatant solution was removed by filtration and the purple solid was washed with Et_2O (10 mL) and residual solvent was removed under vacuum. Single crystals suitable for characterization by X-ray crystallography were obtained by standing the crude reaction mixture in *n*-hexane at room temperature for 3 days. Yield: 0.23 g, 41%. Solid-state $^{31}P\{^1H\}$ CP-MAS NMR: δ -55.9, -77.9. Solid-state $^{29}Si\{^1H\}$-INEPT CP-MAS NMR: δ 111.7.

Synthesis of $[(Mes_2P)_3Si]Li(THF)$ (5.THF): A mixture of $[(Mes_2P)_2Si]_2$ (0.178 g, 0.16 mmol) and $[(Mes)_2P]Li(THF)_2$ (0.132 g, 0.31 mmol) was dissolved in THF (5 mL) stirred for 3 days. The resulting red solution was filtered to remove a small amount of dark solid and the solvent was removed from the filtrate *in vacuo*. Diethyl ether (5 mL) was added to the resulting sticky orange solid to give a yellow solution that rapidly precipitated a yellow solid. The supernatant solution was removed by filtration and the yellow solid was washed with cold (-10 °C) Et_2O (2 mL) and residual solvent was removed *in vacuo*. Yield: 0.20 g, 70%. ^1H NMR [C_6D_6]: δ 1.37 (m, THF, 4H) 2.07 (s, 9H, Me), 2.10 (s, 9H, Me), 2.36 (s, 9H, Me),

2.63 (s, 9H, Me), 2.65 (s, 9H, Me), 2.83 (s, 9H, Me), 3.85 (m, 4H, THF), 6.28 (s, 3H, ArH), 6.35 (s, 3H, ArH), 6.67 (s, 6H, ArH). $^{13}C\{^{1}H\}$ NMR [C_6D_6]: δ 20.84, 20.94, (Me), 24.15 (m, Me), 25.33 (Me), 25.50 (THF) 25.89 (m, Me), 28.05 (Me), 69.59 (THF), 129.01 (ArH), 129.56 (d, J_{PC} = 2.9 Hz, ArH), 130.09, 130.36 (ArH), 133.24 (m, ArH), 133.59 (m, ArH), 135.41, 136.80 (Ar), 143.30 (m, 2Ar), 145.67, 146.34 (Ar). ^{7}Li NMR [C_6D_6]: δ 1.6 (q, J_{LiP} = 42 Hz). $^{29}Si\{^{1}H\}$-INEPT NMR [C_6D_6]: δ 16.1 (q, J_{SiP} = 110 Hz). $^{31}P\{^{1}H\}$ NMR [C_6D_6]: δ -63.1 (q, J_{LiP} = 42 Hz).

X-ray Crystallography: Crystal structure datasets for all compounds were collected on an Xcalibur, Atlas, Gemini ultra diffractometer using an Enhance Ultra X-ray Source ($\lambda_{CuK\alpha}$ = 1.54184 Å) for **4** and **7** and a fine-focus sealed X-ray tube ($\lambda_{MoK\alpha}$ = 0.71073 Å) for **1**. Using an Oxford Cryosystems CryostreamPlus open-flow N_2 cooling device, data for all structures were collected at 150 K. Cell refinement, data collection and data reduction were undertaken using CrysAlisPro.[S3] For all compounds an analytical numeric absorption correction was applied using a multifaceted crystal model based on expressions derived by R. C. Clark and J. S. Reid.[S4] The structures were solved using XT[S5] and refined by XL[S6] through the Olex2 interface.[S7] Hydrogen atoms were positioned with idealized geometry and their displacement parameters were constrained using a riding model. Disorder in the structures was modelled using restraints and constraints where appropriate to maintain a physically meaningful refinement. The structure of **4** contained solvent-accessible voids. The contents of the voids appeared to be molecules of THF disordered across several sites about a three-fold centre of symmetry. No sensible model could be produced for this disorder and so the associated electron density was treated with the Olex2 mask routine.[S7] For **7** **i**dentical disorder was also found for a second crystal obtained from a newly synthesised and crystallised batch of material.

Table S1. Crystallographic data for **1**, **4** and **7**.

Compound	**1**	**4**	**7**
formula	$C_{62}H_{96}Cl_2P_2Si$	$C_{66}H_{90}KO_3P_3Si$	$C_{72}H_{88}P_4Si_2$
M_w	1002.31	1091.47	1133.48
cryst. size (mm)	0.24×0.19×0.15	0.22×0.18×0.04	0.29×0.13×0.11
cryst. syst.	monoclinic	trigonal	triclinic
space group	$P2_1/n$	$R3c$	P-1
a (Å)	14.2776(3)	18.48450(14)	11.2076(4)
b (Å)	13.4893(3)	18.48450(14)	12.3993(5)
c (Å)	30.8896(7)	33.0361(3)	13.0756(5)
α (deg)			108.421(4)
β (deg)	91.8809(18)		107.709(4)
γ (deg)			102.327(3)
V (Å^3)	5946.0(2)	9775.40(17)	1542.91(11)
Z	4	6	1
μ (mm^{-1})	0.219	1.897	1.814
reflns. measd.	39804	50107	21948
unique reflns.	13085	3824	5471

R_{int}	0.053	0.038	0.035
refined parameters	666	244	367
R (on F, $F^2 > 2\sigma$)[a]	0.051	0.028	0.044
R_w (on F^2, all data)[a]	0.113	0.074	0.130
goodness of fit[a]	1.012	1.040	1.033
max, min electron density (e Å^{-3})	0.39,-0.36	0.14, -0.15	0.70, -0.55

[a] $R = \Sigma||F_o|-|F_c||/\Sigma|F_o|$; $R_w = [\Sigma w(F_o^2-F_c^2)^2/\Sigma w(F_o^2)^2]^{1/2}$; $S = [\Sigma w(F_o^2-F_c^2)^2/(\text{no. data - no. params})]^{1/2}$ for all data.

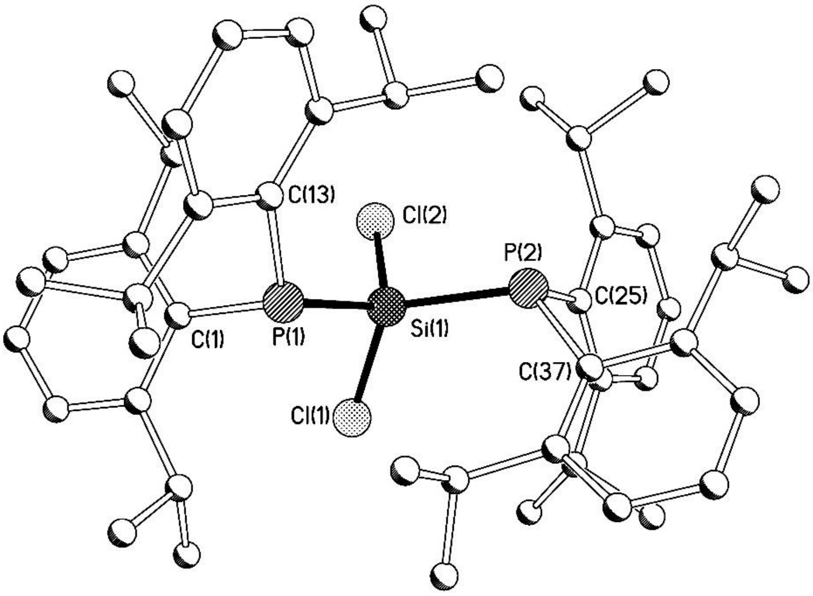

Figure S1. Molecular structure of **1**.$(C_7H_{14})_2$ with H atoms and solvent of crystallization omitted for clarity. Selected bond lengths (Å) and angles (°): Si(1)-P(1) 2.2448(7), Si(1)-P(2) 2.2483(7), Si(1)-Cl(1) 2.0654(8), Si(1)-Cl(2) 2.0592(7), P(1)-C(13) 1.861(2), P(1)-C1) 1.864(2), P(2)-C(25) 1.8638(19), P(2)-C37) 1.857(2), P(1)-Si(1)-P(2) 116.67(3), Cl(1)-Si(1)-Cl(2) 101.95(3).

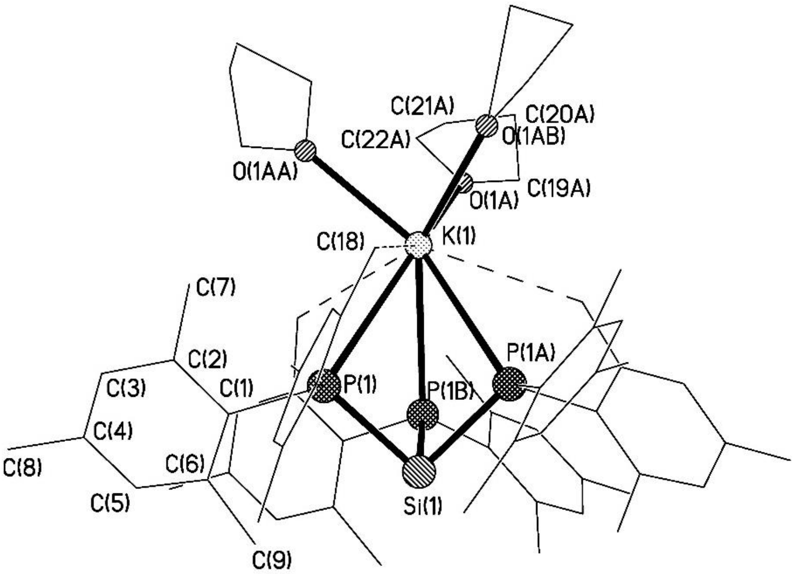

Figure S2. Molecular structure of **4** with H atoms and minor disorder components omitted for clarity. Selected bond lengths (Å) and angles (°): K(1)-P(1) 3.4494(8), K(1)-O(1A) 2.780(14), K(1)…C(18) 3.362(3), Si(1)-P(1) 2.3972(8), C(1)-P(1) 1.870(2), C(10)-P(1) 1.866(2), P(1)-K(1)-P(1A) 58.01(2), P(1)-Si(1)-P(1A) 88.49(3), Si|(1)-P(1)-K(1) 92.28(3).

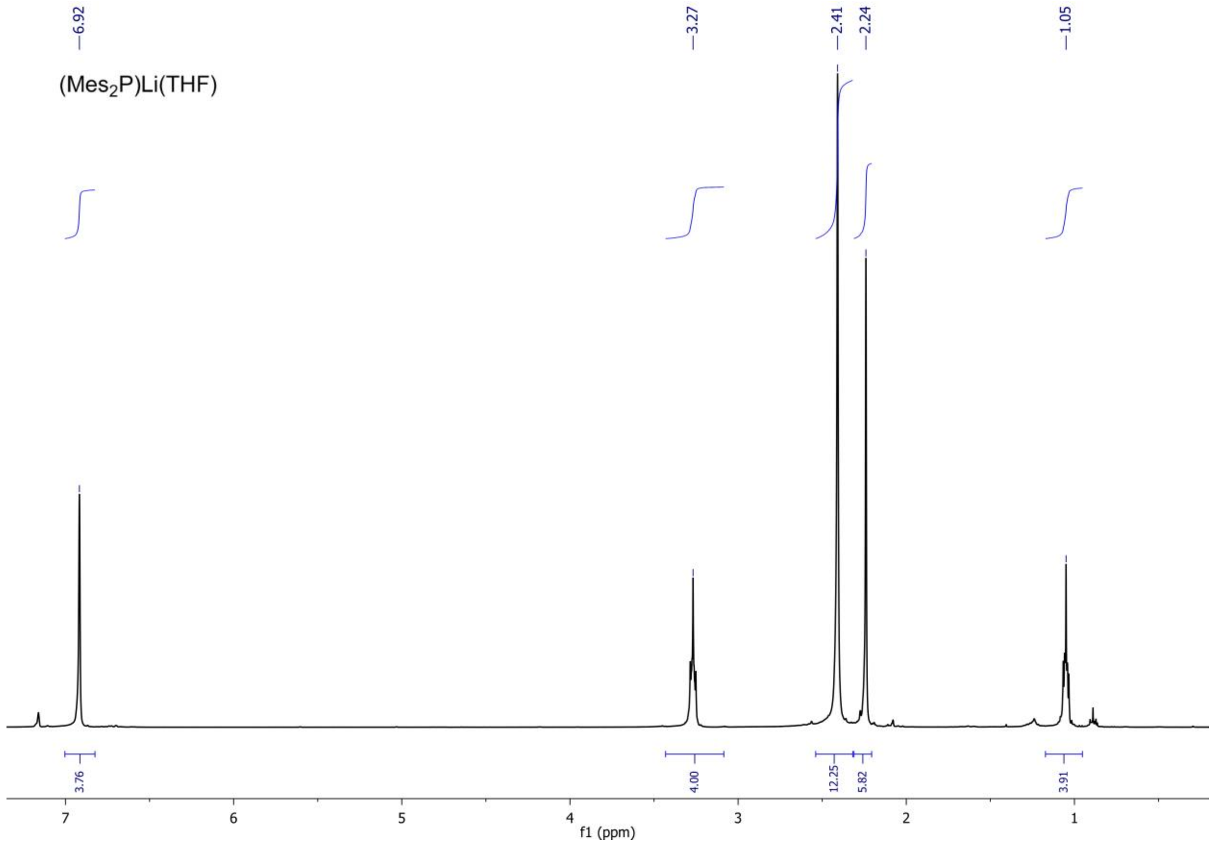

^{1}H NMR spectrum of $[(Mes)_2P]Li(THF)$ in C_6D_6.

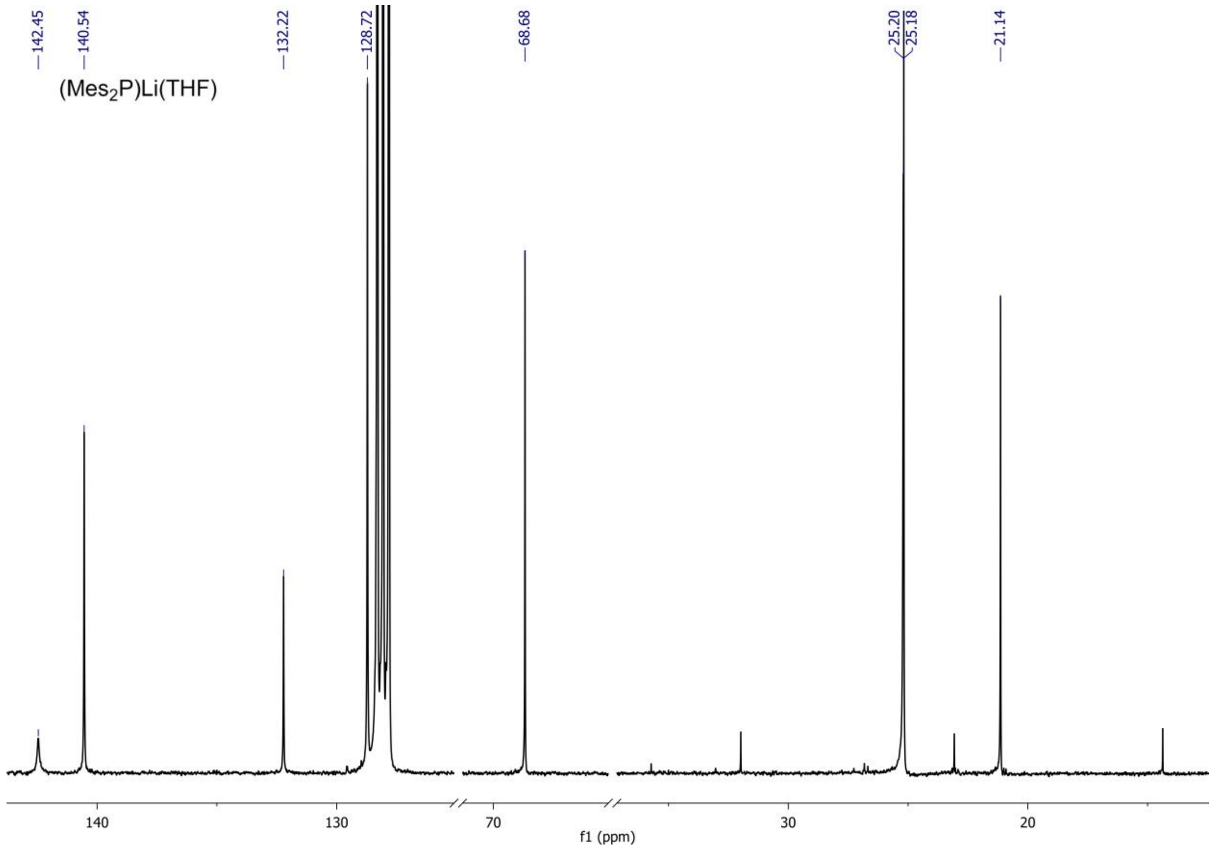

$^{13}C\{^{1}H\}$ NMR spectrum of $[(Mes)_2P]Li(THF)$ in C_6D_6.

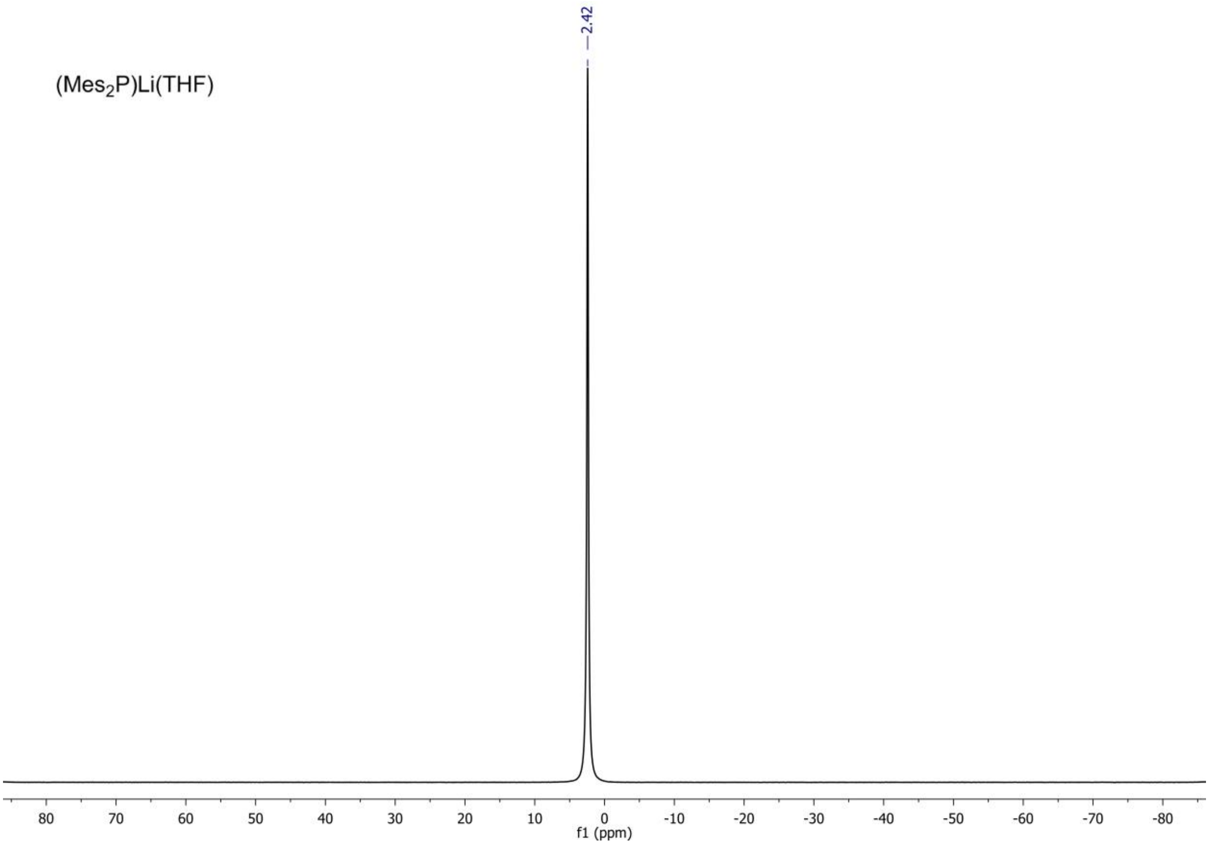

$^{7}Li\{^{1}H\}$ NMR spectrum of $[(Mes)_2P]Li(THF)$ in C_6D_6.

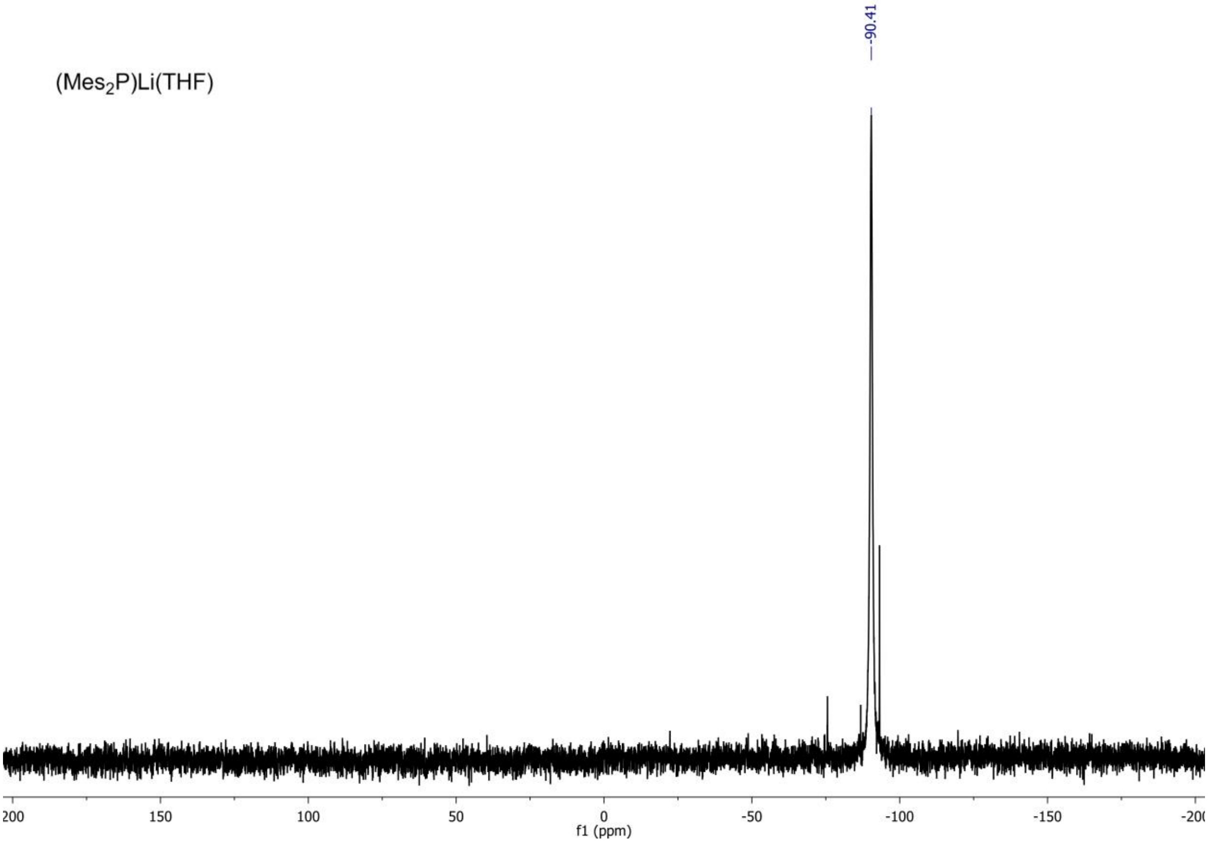

$^{31}P\{^{1}H\}$ NMR spectrum of $[(Mes)_2P]Li(THF)$ in C_6D_6.

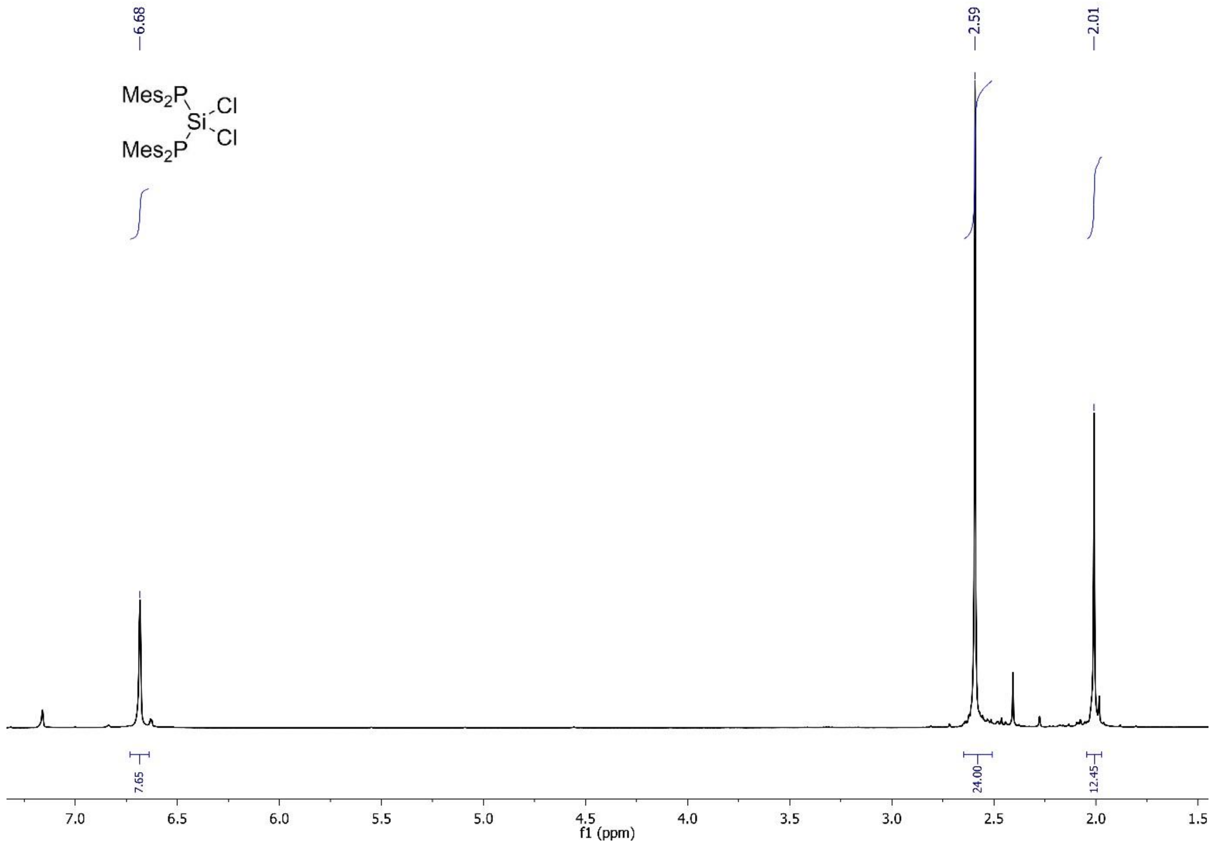

^{1}H NMR spectrum of **2** in $CDCl_3$.

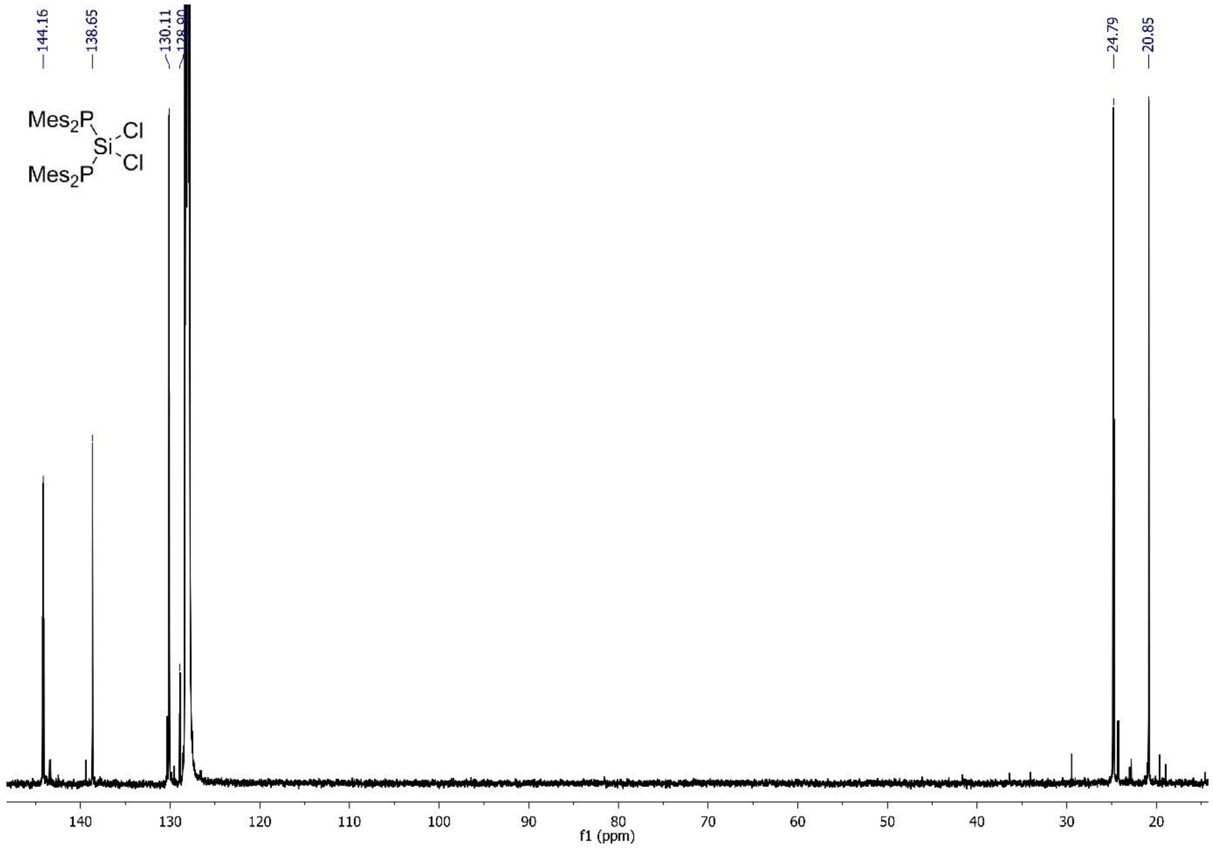

$^{13}C\{^{1}H\}$ NMR spectrum of **2** in $CDCl_3$

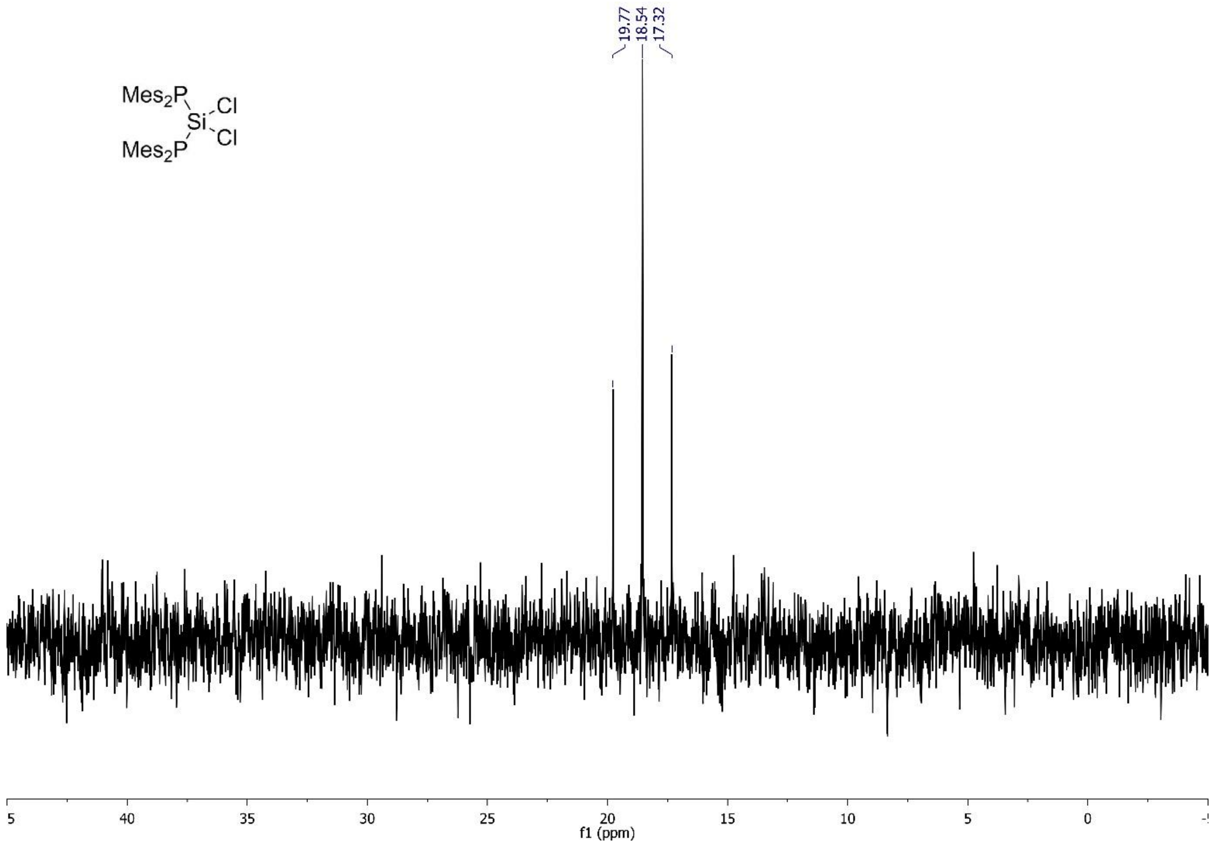

$^{29}Si\{^{1}H\}$ INEPT NMR spectrum of **2** in $CDCl_3$

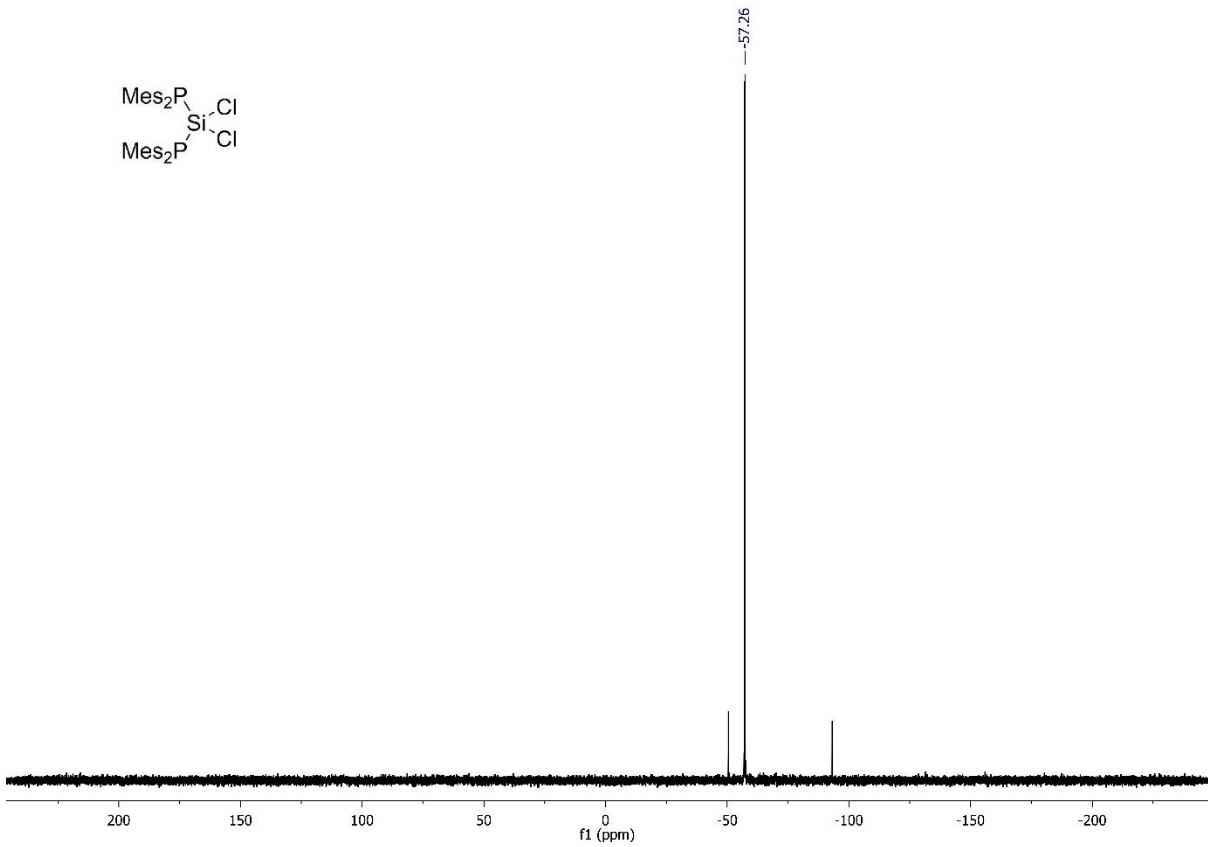

$^{31}P\{^{1}H\}$ NMR spectrum of **2** in $CDCl_3$

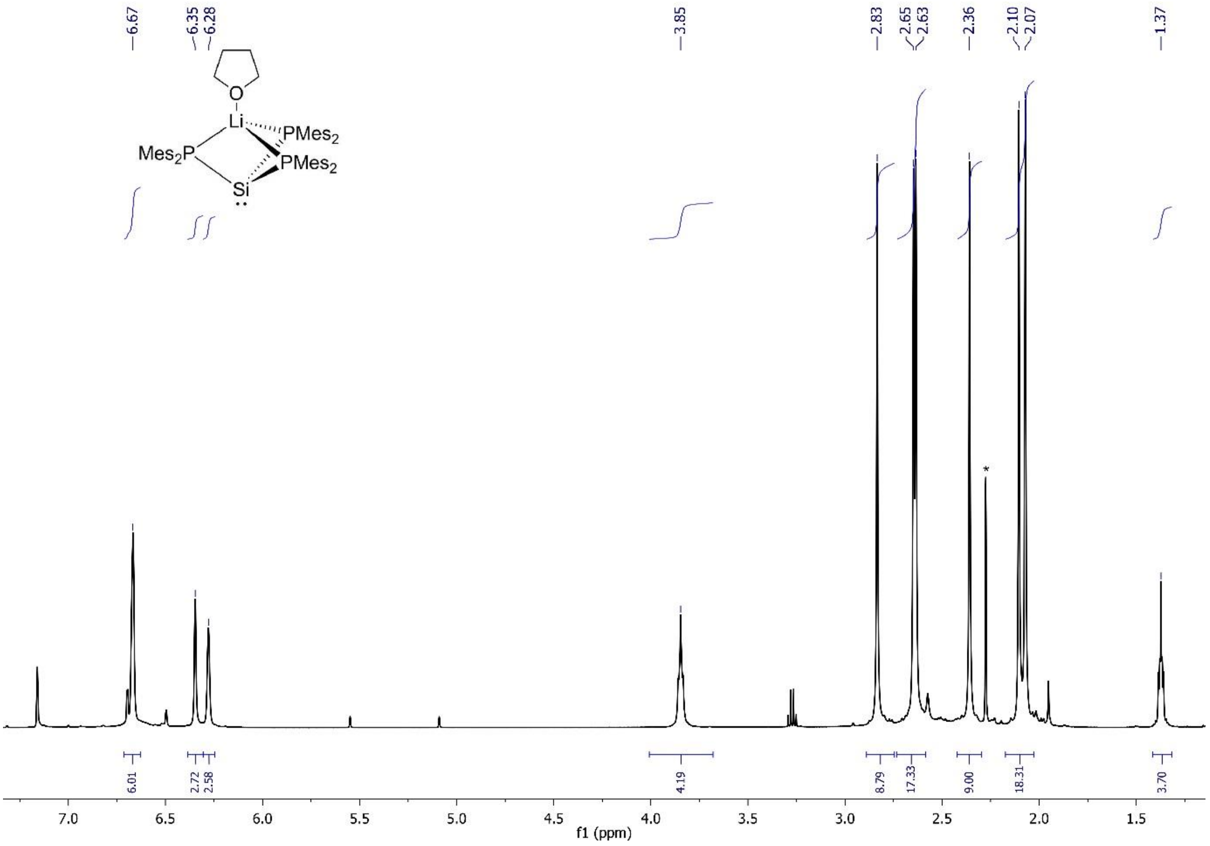

^{1}H NMR spectrum of **5.THF** in C_6D_6. [*residual signals from $(Mes)_2PH$]

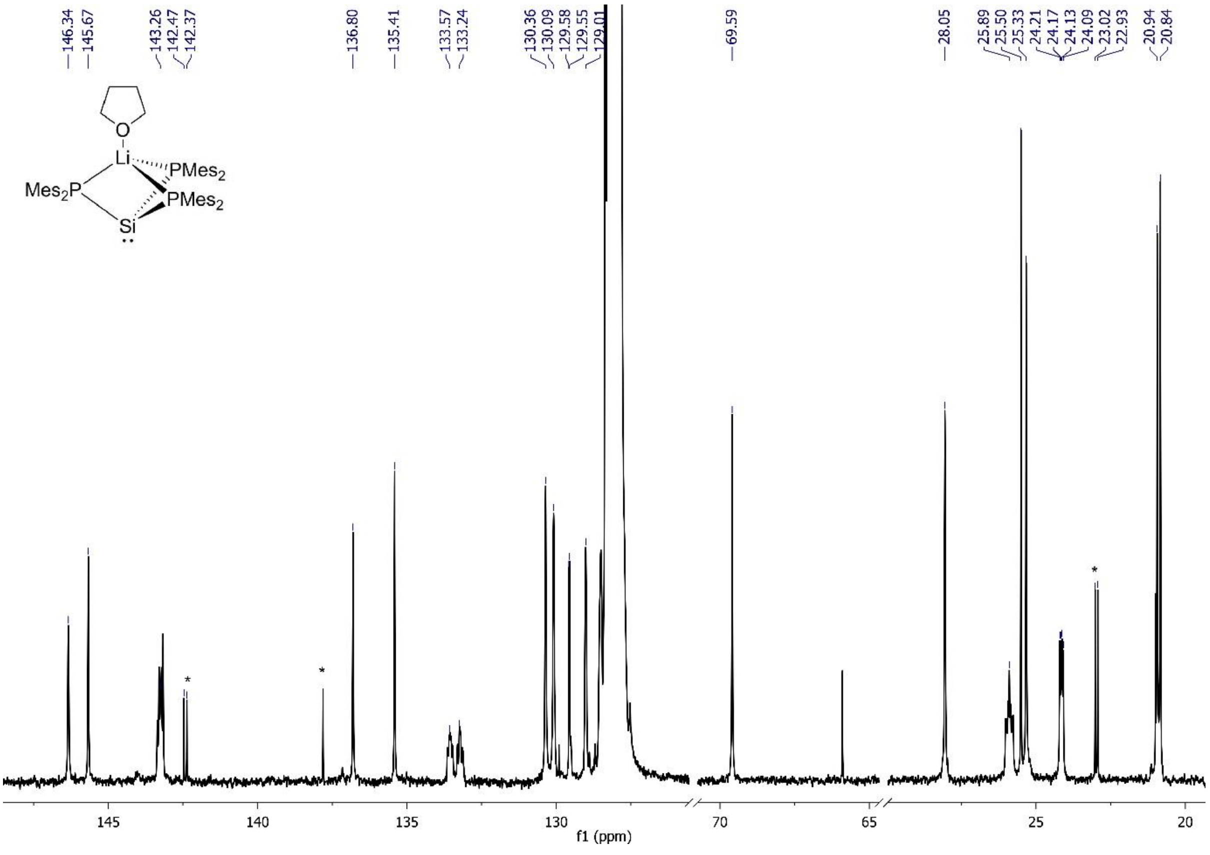

^{13}C{^{1}H} NMR spectrum of **5.THF** in C_6D_6 [*residual signals from $(Mes)_2PH$]

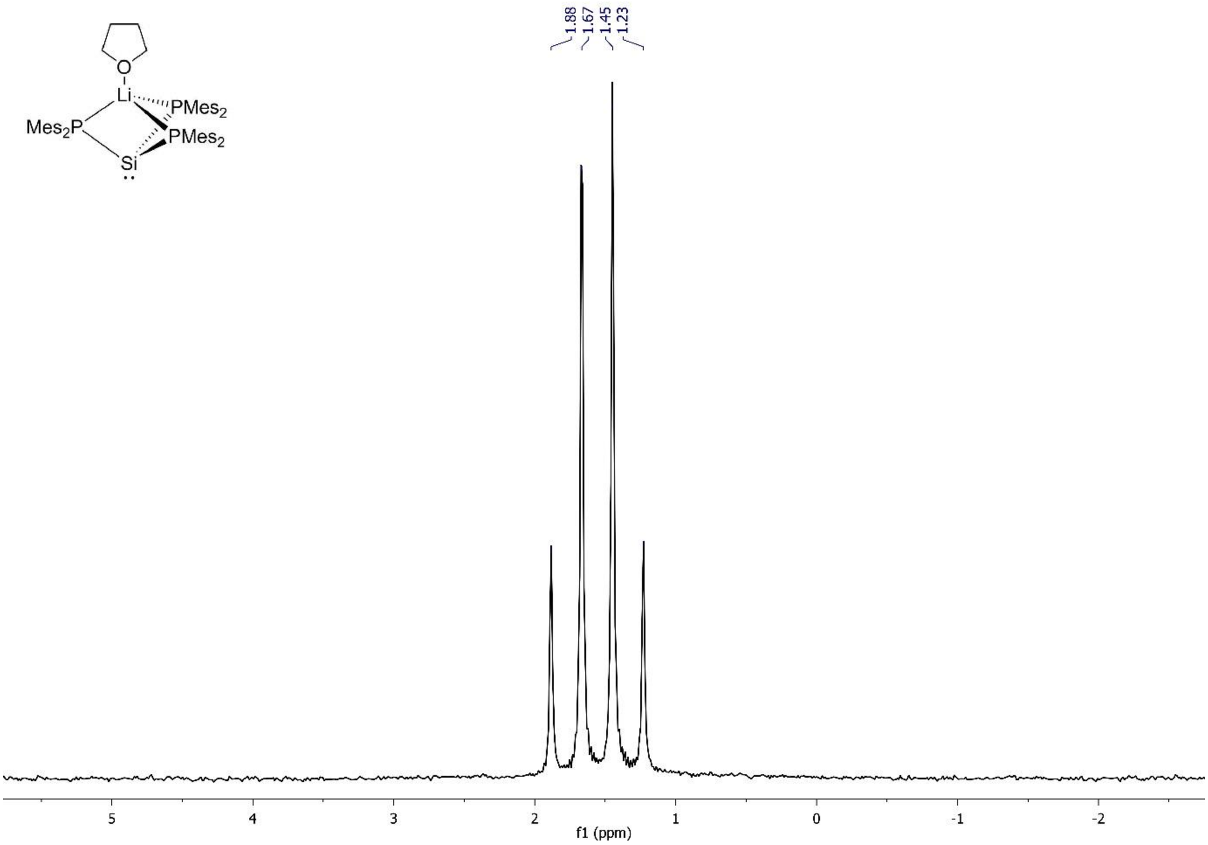

$^{7}Li\{^{1}H\}$ NMR spectrum of **5.THF** in C_6D_6

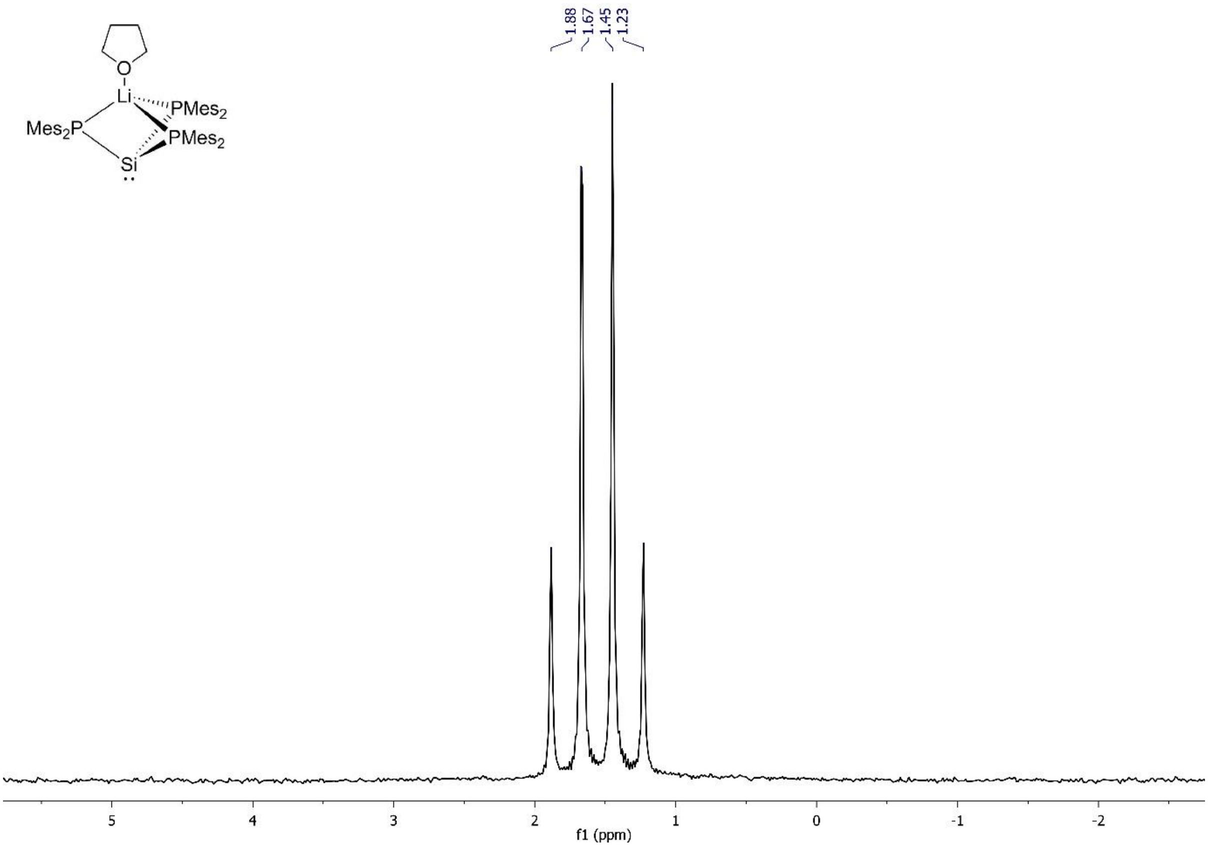

$^{29}Si\{^{1}H\}$ INEPT NMR spectrum of **5.THF** in C_6D_6

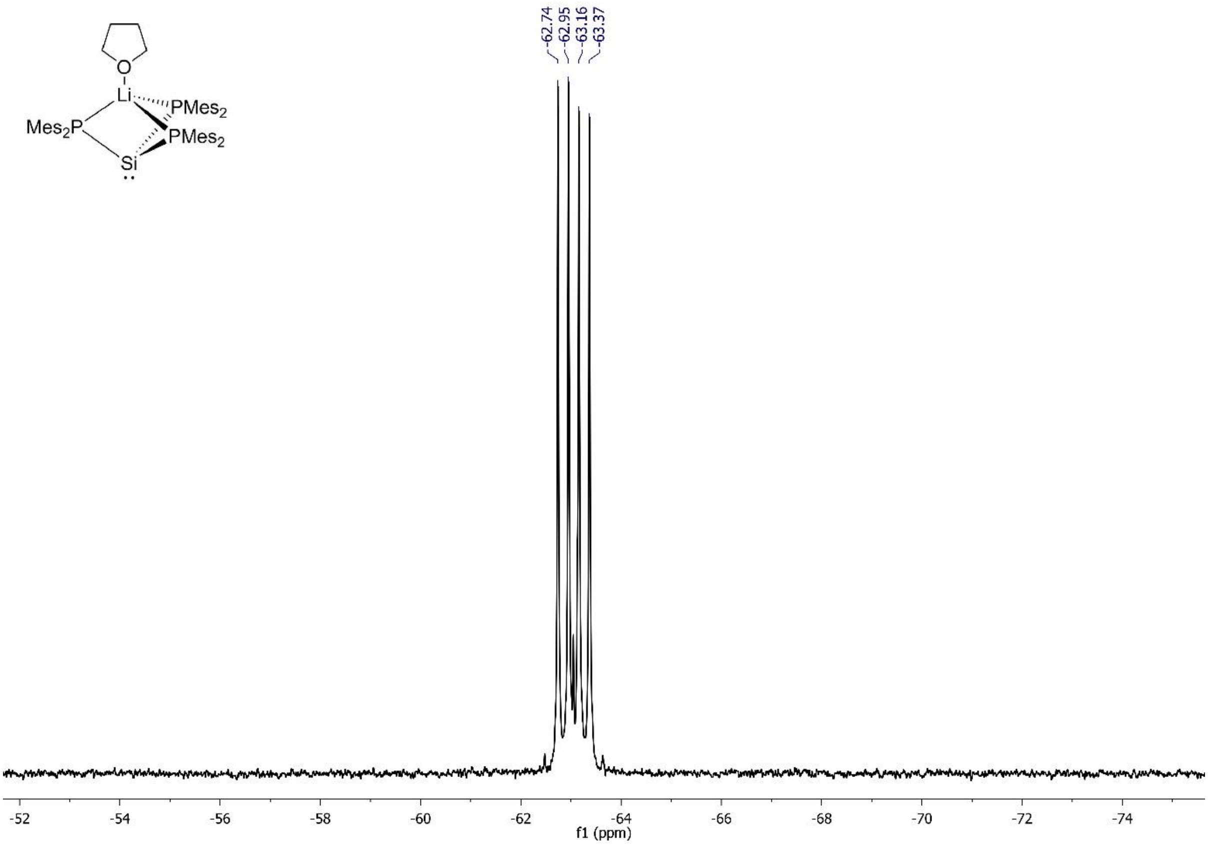

$^{31}P\{^{1}H\}$ NMR spectrum of **5.THF** in C_6D_6

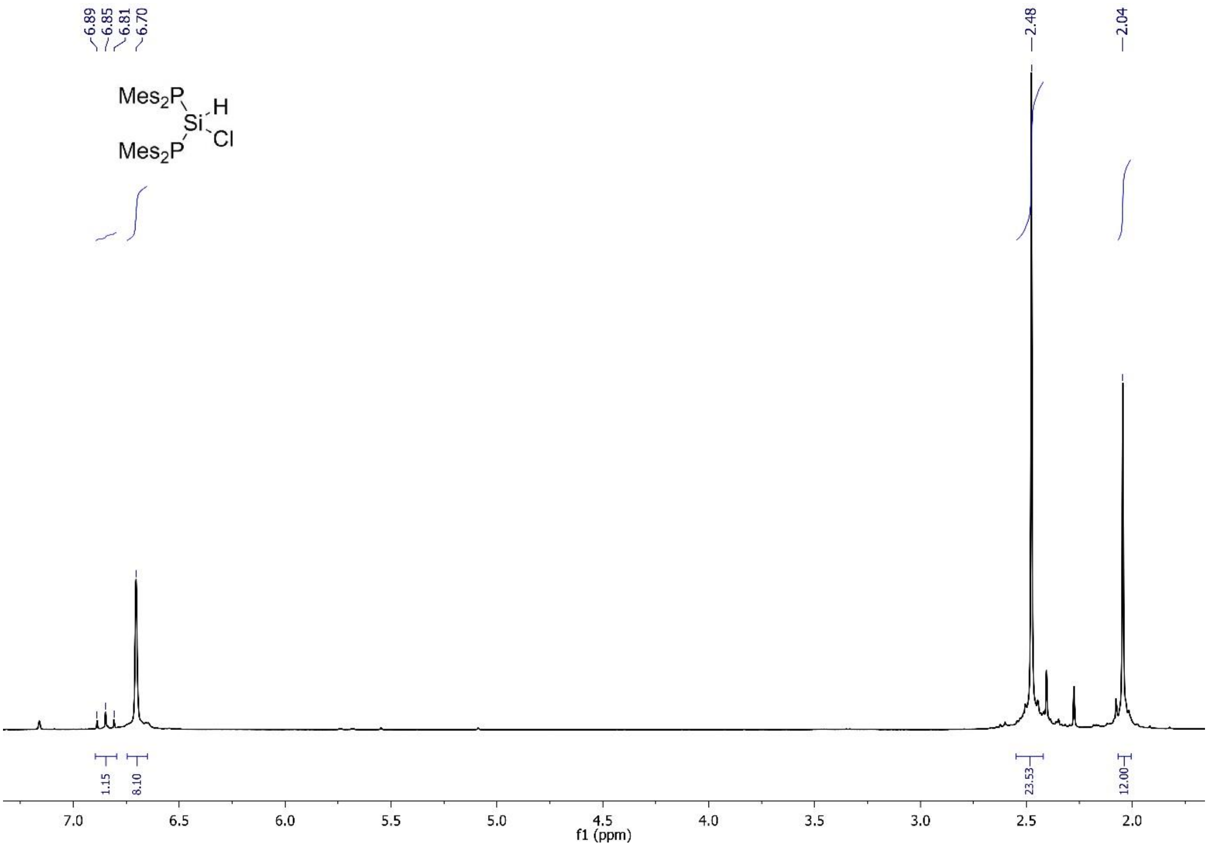

^{1}H NMR spectrum of **6** in C_6D_6

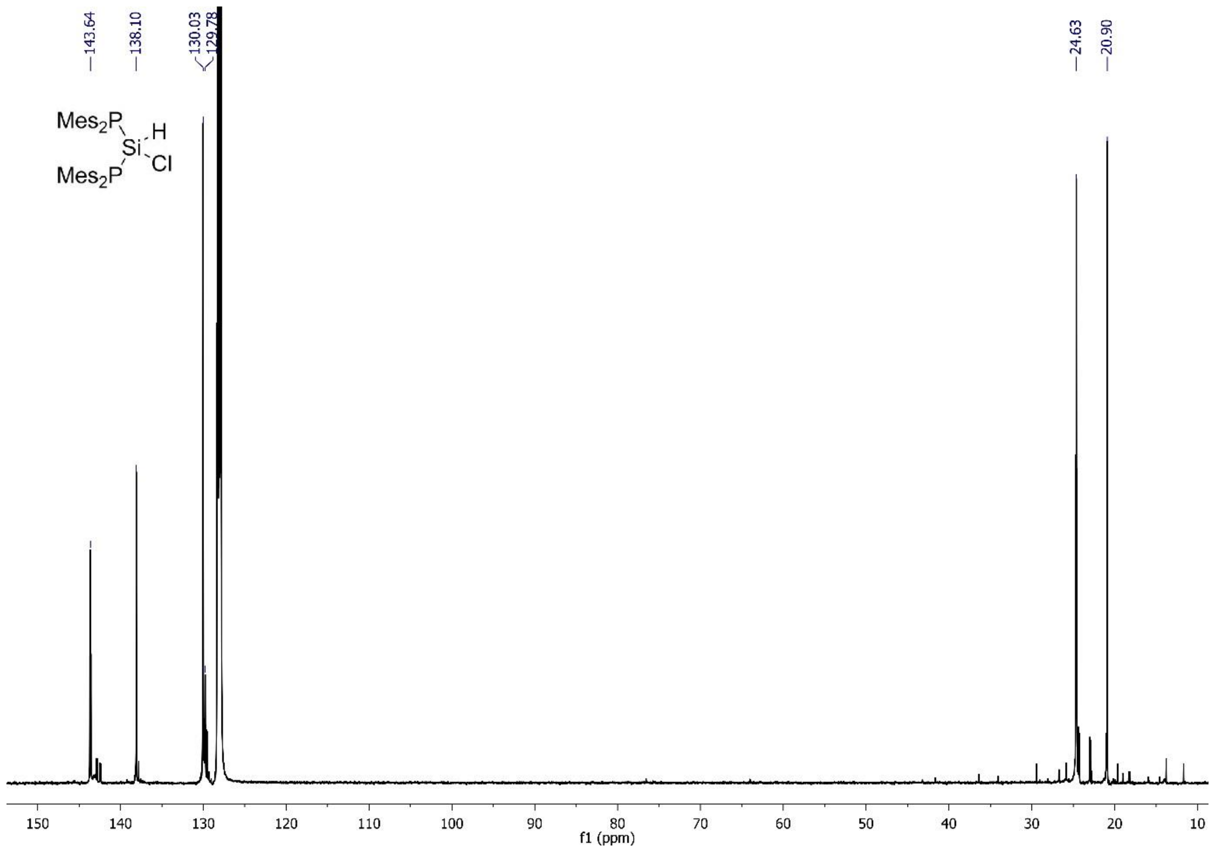

$^{13}C\{^{1}H\}$ NMR spectrum of **6** in C_6D_6

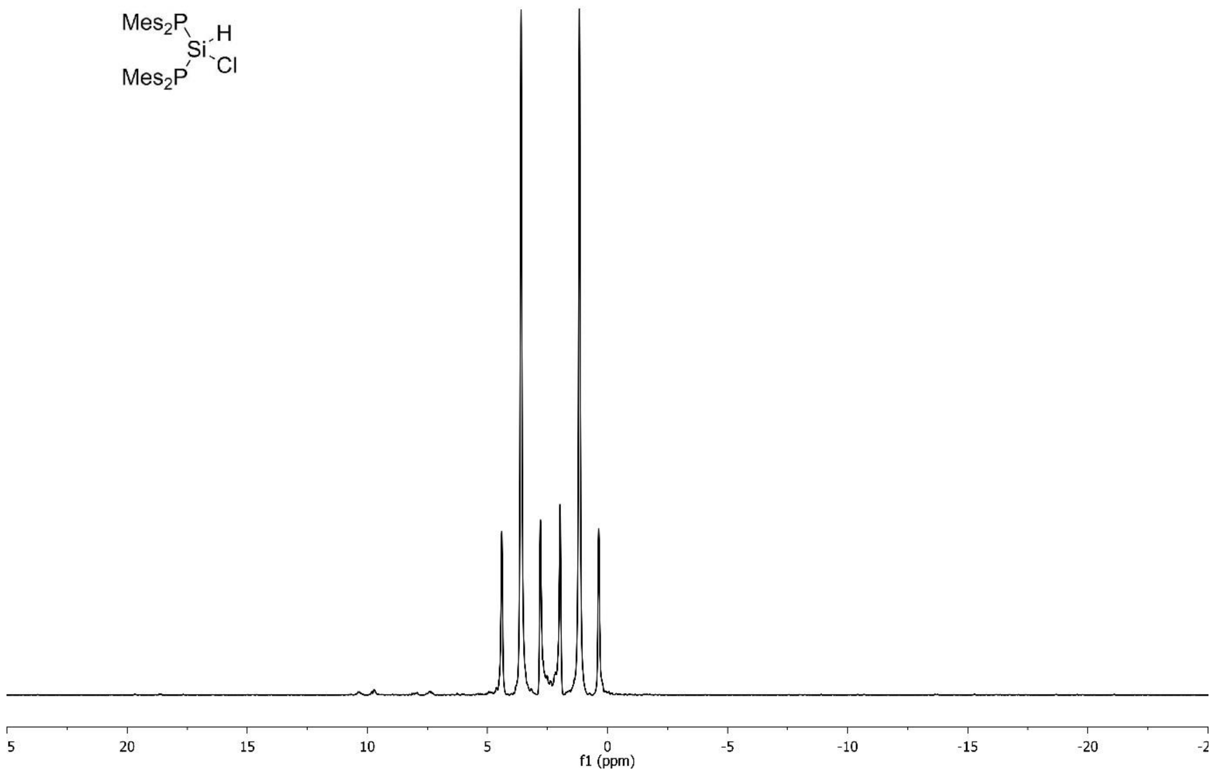

^{29}Si INEPT spectrum of **6** in C_6D_6

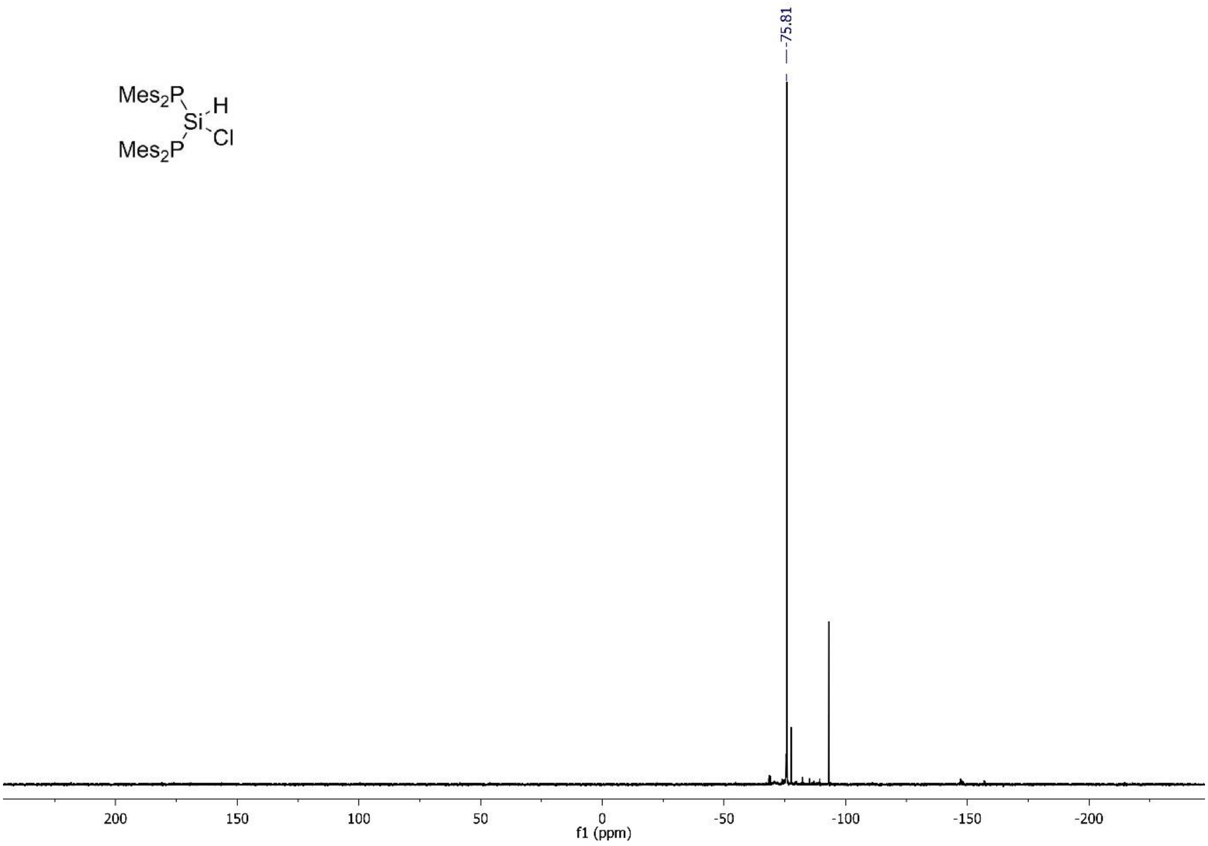

$^{31}P\{^{1}H\}$ NMR spectrum of **6** in C_6D_6

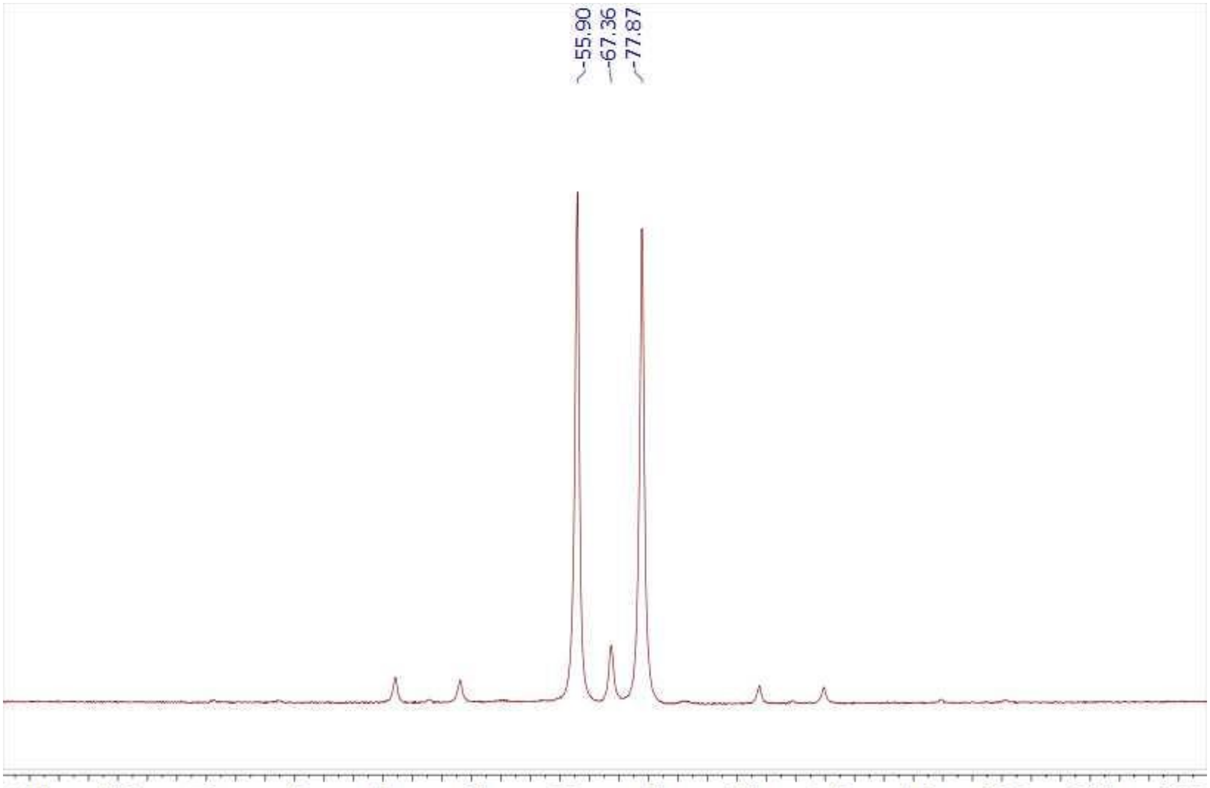

$^{31}P\{^{1}H\}$ solid-state CP-MAS NMR spectrum of **7**.

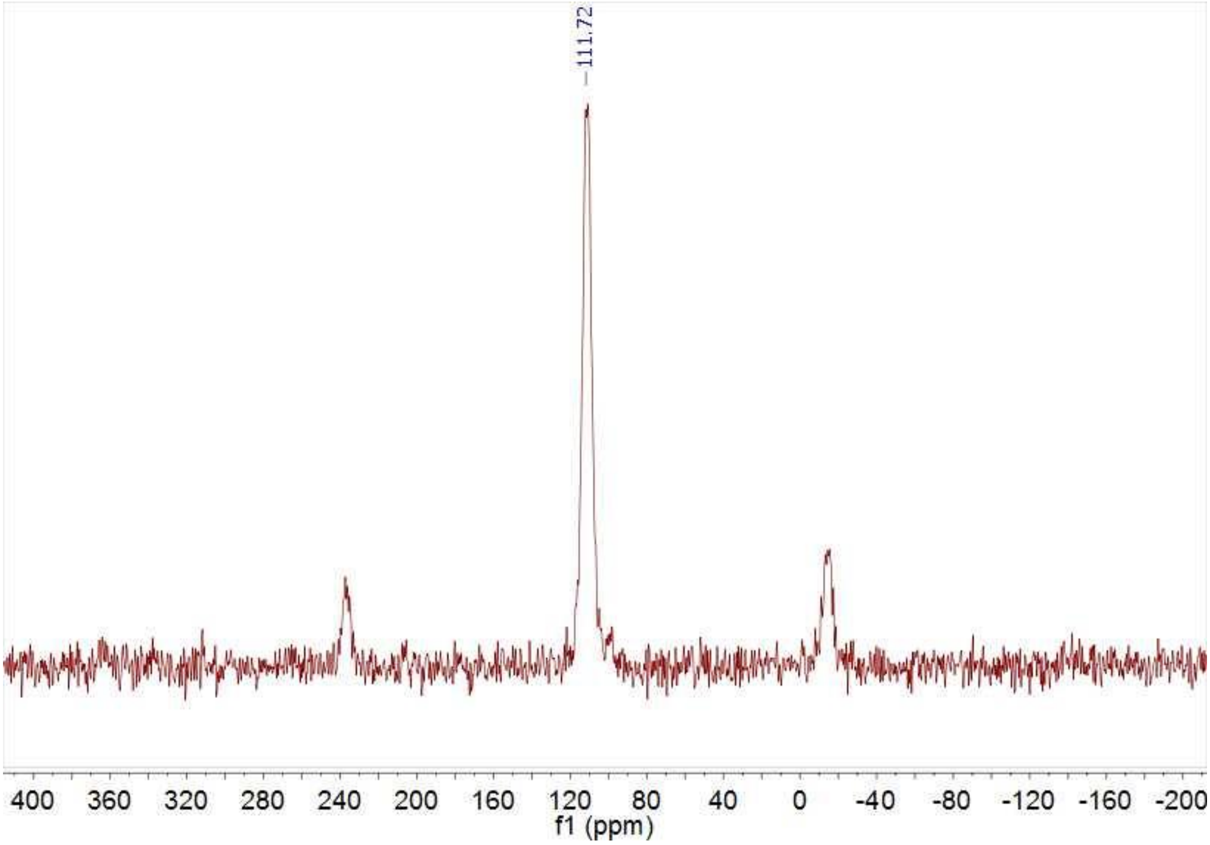

$^{29}Si\{^{1}H\}$ solid-state CP-MAS NMR spectrum of **7**.

DFT Calculations: Geometry optimizations were performed with the Gaussian09 suite of programs (revision E.01).[S8] The pure B97D functional,[S9] which includes a correction for dispersion effects, was employed throughout; the 6-311G(2d,p) all-electron basis set[S10] was used on all atoms. The identity of minima was confirmed by the absence of imaginary vibrational frequencies in each case. Automatic density fitting was employed for all geometry optimizations and frequency calculations. Calculation of the dimerization energy of $\mathbf{3}_{\mathbf{plan}}$ was corrected for Basis Set Superposition Error using Boys and Bernadi's Counterpoise method.[S11] NMR shielding tensors were calculated using the GIAO method[S12] at the B97D/6-311++G(2d,p)//6-311G(2d,p) level of theory, chemical shifts are quoted in ppm relative to H_3PO_4 (based on PMe_3 at -61 ppm calculated at the same level of

theory). Natural Bond Orbital analyses were performed using the NBO 3.1 module of Gaussian09.[S13]

Table S3. Calculated ^{31}P and ^{29}Si NMR chemical shifts for **3$_{plan}$**, **3$_{pyr}$**, **7$_{maj}$**, **7$_{min}$**, and **7$_{alt}$**.

	**3$_{plan}$**	**3$_{pyr}$**	**7$_{maj}$**	**7$_{min}$**	**7$_{alt}$**
^{31}P	18.9<br>18.9	-35.4<br>-35.4	-28.7<br>-41.5<br>-57.2<br>-61.8	-53.6<br>-59.1	-27.9<br>-31.9<br>-35.6<br>-42.7
^{29}Si	164.7	202.6	168.7<br>112.8	103.1	-9.9<br>61.5

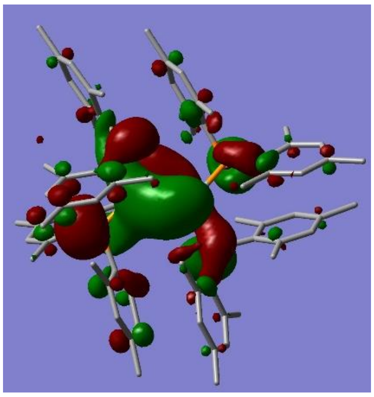

HOMO of **7$_{maj}$**

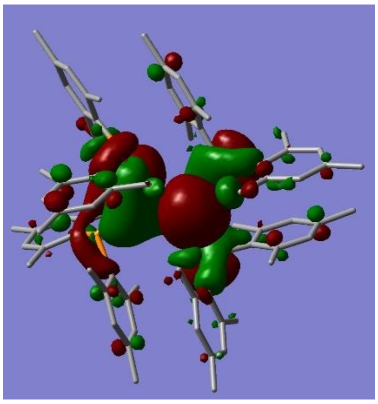

LUMO of **7$_{maj}$**

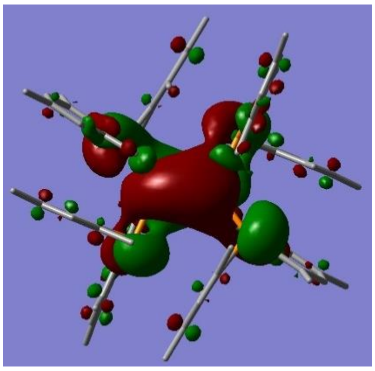

HOMO of **7$_{min}$**

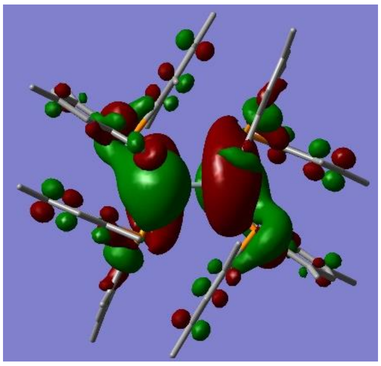

LUMO of **7$_{min}$**

Figure S3. HOMOs and LUMOs of **7$_{maj}$** and **7$_{min}$**.

Final atomic coordinates and electronic energies:

3plan

Center Number	Atomic Number	Atomic Type	Coordinates (Angstroms) X	Y	Z
1	15	0	1.660682	-0.896649	-0.519190
2	15	0	-1.660849	-0.897545	0.520501
3	6	0	3.354042	-1.525455	-0.202114
4	6	0	3.708004	-2.335322	0.909793
5	6	0	5.042171	-2.733138	1.062729
6	1	0	5.306791	-3.345125	1.925780
7	6	0	6.044843	-2.358695	0.164230
8	6	0	5.682228	-1.553329	-0.918661
9	1	0	6.446764	-1.242146	-1.631436
10	6	0	4.362925	-1.134765	-1.125762
11	6	0	2.727016	-2.775934	1.973780
12	1	0	2.030789	-1.970248	2.240504
13	6	0	7.469410	-2.829075	0.346918
14	1	0	7.615698	-3.820412	-0.108742
15	6	0	4.071733	-0.263694	-2.329712
16	1	0	3.806804	0.759914	-2.032014
17	6	0	1.698489	0.933949	-0.423674
18	6	0	2.457895	1.636121	0.549064
19	6	0	2.471918	3.034749	0.512082
20	1	0	3.053809	3.567963	1.265348
21	6	0	1.759274	3.765875	-0.442588
22	6	0	1.015688	3.057873	-1.388316
23	1	0	0.444787	3.605432	-2.137568
24	6	0	0.975942	1.660676	-1.404821
25	6	0	3.263974	0.950209	1.628946
26	1	0	4.217429	0.571924	1.237723
27	6	0	1.779695	5.276144	-0.426607
28	1	0	2.806704	5.657929	-0.344042
29	6	0	0.182717	0.970054	-2.488697
30	1	0	-0.560656	0.289311	-2.061010
31	6	0	-3.354315	-1.525801	0.202576
32	6	0	-3.708177	-2.335991	-0.909132
33	6	0	-5.042509	-2.732971	-1.062704
34	1	0	-5.307013	-3.345181	-1.925632
35	6	0	-6.045479	-2.357441	-0.164983
36	6	0	-5.682944	-1.551902	0.917790
37	1	0	-6.447684	-1.239926	1.630000
38	6	0	-4.363480	-1.134109	1.125496
39	6	0	-2.726824	-2.777725	-1.972288
40	1	0	-2.030562	-1.972246	-2.239608
41	6	0	-7.470267	-2.826883	-0.348369
42	1	0	-7.617399	-3.818204	0.107056
43	6	0	-4.072550	-0.262662	2.329241
44	1	0	-3.807431	0.760813	2.031254
45	6	0	-1.698258	0.933102	0.424057
46	6	0	-2.457448	1.635107	-0.548979
47	6	0	-2.471297	3.033747	-0.512423
48	1	0	-3.052993	3.566800	-1.265955
49	6	0	-1.758698	3.765082	0.442119
50	6	0	-1.015266	3.057274	1.388101
51	1	0	-0.444336	3.604985	2.137218
52	6	0	-0.975682	1.660072	1.405008
53	6	0	-3.263394	0.949033	-1.628863

54	1	0	-4.217073	0.571140	-1.237818
55	6	0	-1.779040	5.275348	0.425747
56	1	0	-1.216307	5.664652	-0.434923
57	6	0	-0.182489	0.969725	2.489085
58	1	0	0.560910	0.288884	2.061560
59	14	0	0.000238	-2.255307	-0.000221
60	1	0	-1.327754	5.688353	1.336388
61	1	0	-2.806122	5.657180	0.344238
62	1	0	-4.948151	-0.216073	2.988012
63	1	0	-3.220385	-0.655196	2.901652
64	1	0	-7.724848	-2.914708	-1.412767
65	1	0	-8.179984	-2.136629	0.125290
66	1	0	-2.111483	-3.620050	-1.628436
67	1	0	-3.262674	-3.097162	-2.875052
68	1	0	-0.340486	1.705371	-3.111361
69	1	0	0.841601	0.364138	-3.127460
70	1	0	1.329430	5.688935	-1.337852
71	1	0	1.216006	5.665707	0.433323
72	1	0	3.478903	1.653320	2.443064
73	1	0	2.731683	0.087975	2.043472
74	1	0	0.340704	1.705203	3.111568
75	1	0	-0.841356	0.363957	3.128004
76	1	0	-3.477872	1.651923	-2.443292
77	1	0	-2.731200	0.086511	-2.042920
78	1	0	2.111613	-3.618666	1.631031
79	1	0	3.263231	-3.094402	2.876673
80	1	0	4.947120	-0.217435	-2.988791
81	1	0	3.219338	-0.656364	-2.901692
82	1	0	7.724399	-2.917234	1.411188
83	1	0	8.179364	-2.139207	-0.126950

Final electronic energy (au): -2369.93228475

NIMAG = 0

3$_{\mathbf{pyr}}$:

Center Number	Atomic Number	Atomic Type	Coordinates (Angstroms) X	Y	Z
1	15	0	-1.732100	0.202906	0.958633
2	15	0	1.732102	-0.202930	0.958628
3	6	0	-3.333480	-0.179825	0.104569
4	6	0	-4.455566	0.662462	0.323113
5	6	0	-5.688954	0.326846	-0.251664
6	1	0	-6.540903	0.986621	-0.081187
7	6	0	-5.865717	-0.830161	-1.013232
8	6	0	-4.766824	-1.682434	-1.163570
9	1	0	-4.888193	-2.614217	-1.718139
10	6	0	-3.512237	-1.388732	-0.617644
11	6	0	-4.385033	1.912400	1.174768
12	1	0	-3.859045	2.730145	0.663737
13	6	0	-2.399459	-2.398769	-0.809276
14	1	0	-1.854043	-2.597805	0.121542
15	6	0	-1.307035	1.980410	0.536085
16	6	0	-0.612278	2.712878	1.551902

17	6	0	-0.165894	4.006388	1.286769
18	1	0	0.360947	4.548036	2.072971
19	6	0	-0.372873	4.629880	0.047963
20	6	0	-1.085284	3.929982	-0.922055
21	1	0	-1.278852	4.404968	-1.884143
22	6	0	-1.567254	2.626010	-0.714394
23	6	0	-0.364277	2.140647	2.929962
24	1	0	0.328697	1.289888	2.889770
25	6	0	-2.407051	2.025687	-1.822298
26	1	0	-2.227955	0.956735	-1.965282
27	6	0	3.333468	0.179836	0.104557
28	6	0	4.455575	-0.662427	0.323097
29	6	0	5.688957	-0.326776	-0.251667
30	1	0	6.540921	-0.986530	-0.081190
31	6	0	5.865696	0.830246	-1.013222
32	6	0	4.766783	1.682492	-1.163560
33	1	0	4.888135	2.614281	-1.718119
34	6	0	3.512198	1.388753	-0.617645
35	6	0	4.385064	-1.912376	1.174738
36	1	0	3.859082	-2.730121	0.663703
37	6	0	2.399396	2.398763	-0.809280
38	1	0	1.853993	2.597810	0.121543
39	6	0	1.307057	-1.980437	0.536068
40	6	0	0.612288	-2.712916	1.551879
41	6	0	0.165889	-4.006410	1.286730
42	1	0	-0.360972	-4.548053	2.072922
43	6	0	0.372874	-4.629895	0.047914
44	6	0	1.085277	-3.929991	-0.922095
45	1	0	1.278815	-4.404958	-1.884197
46	6	0	1.567264	-2.626021	-0.714418
47	6	0	0.364287	-2.140692	2.929943
48	1	0	-0.328669	-1.289917	2.889756
49	6	0	2.407050	-2.025691	-1.822326
50	1	0	2.227972	-0.956734	-1.965286
51	14	0	-0.000005	-0.000049	-0.597219
52	1	0	3.476681	-2.131979	-1.593049
53	1	0	2.203396	-2.543255	-2.767559
54	1	0	-2.807243	-3.349838	-1.174108
55	1	0	-1.657928	-2.052051	-1.543438
56	1	0	-3.476679	2.131949	-1.593000
57	1	0	-2.203422	2.543271	-2.767525
58	1	0	5.395224	-2.258656	1.425767
59	1	0	3.837099	-1.725826	2.108829
60	1	0	2.807159	3.349832	-1.174140
61	1	0	1.657860	2.052016	-1.543421
62	1	0	-0.066250	-2.905597	3.588188
63	1	0	1.298389	-1.770606	3.374286
64	1	0	-5.395187	2.258688	1.425809
65	1	0	-3.837063	1.725832	2.108852
66	1	0	0.066241	2.905555	3.588218
67	1	0	-1.298373	1.770536	3.374297
68	6	0	-7.197330	-1.155703	-1.650708
69	1	0	-7.205064	-0.870704	-2.714072
70	1	0	-7.409304	-2.232210	-1.600258
71	1	0	-8.015905	-0.617077	-1.156387
72	6	0	7.197304	1.155814	-1.650694
73	1	0	7.205199	0.870411	-2.713949
74	1	0	7.409059	2.232381	-1.600627
75	1	0	8.015940	0.617533	-1.156096
76	6	0	0.180279	6.008561	-0.220806
77	1	0	1.271624	5.967371	-0.357206

78	1	0	-0.256553	6.444372	-1.127696
79	1	0	-0.015505	6.685276	0.622167
80	6	0	-0.180184	-6.008630	-0.220781
81	1	0	0.255226	-6.443666	-1.128724
82	1	0	0.017425	-6.685823	0.621392
83	1	0	-1.271790	-5.967835	-0.355150

Final electronic energy (au): -2369.92570105

NIMAG = 0

7_{maj}:

Center Number	Atomic Number	Atomic Type	Coordinates (Angstroms) X	Y	Z
1	15	0	2.221624	-1.342845	1.110898
2	15	0	2.030066	2.127898	-0.447118
3	14	0	1.187690	0.019818	-0.414032
4	6	0	3.788396	-2.180792	0.571776
5	6	0	4.424254	-2.951256	1.585455
6	6	0	5.611759	-3.634931	1.292324
7	1	0	6.090762	-4.208199	2.087206
8	6	0	6.188469	-3.617858	0.020939
9	6	0	5.527481	-2.896332	-0.975685
10	1	0	5.946467	-2.876248	-1.982555
11	6	0	4.347282	-2.184306	-0.729779
12	6	0	3.871519	-3.078796	2.990388
13	1	0	3.953763	-2.138265	3.550475
14	1	0	4.411330	-3.858539	3.541908
15	1	0	2.804442	-3.341364	2.966074
16	6	0	7.445500	-4.399625	-0.282716
17	1	0	7.201260	-5.412010	-0.640798
18	1	0	8.071937	-4.508134	0.612052
19	1	0	8.037901	-3.908651	-1.065856
20	6	0	3.732950	-1.438025	-1.885496
21	1	0	2.706268	-1.778875	-2.067729
22	1	0	4.311618	-1.597535	-2.804096
23	1	0	3.700897	-0.354545	-1.702880
24	6	0	2.785150	-0.130339	2.402984
25	6	0	1.954181	0.005768	3.549074
26	6	0	2.324401	0.885448	4.572647
27	1	0	1.671393	0.985102	5.440580
28	6	0	3.498227	1.639533	4.510138
29	6	0	4.309246	1.491005	3.381763
30	1	0	5.227056	2.076366	3.308199
31	6	0	3.980652	0.631853	2.325679
32	6	0	0.661778	-0.763316	3.698169
33	1	0	-0.017278	-0.553517	2.859228
34	1	0	0.153199	-0.491331	4.631636
35	1	0	0.835684	-1.847153	3.691488
36	6	0	3.855192	2.622175	5.600959
37	1	0	3.485025	3.629372	5.353895
38	1	0	4.942862	2.695605	5.731046
39	1	0	3.407769	2.331384	6.560241
40	6	0	4.933043	0.567189	1.154028

41	1	0	5.568074	-0.327575	1.199984
42	1	0	5.582402	1.451485	1.145841
43	1	0	4.391863	0.529633	0.204226
44	6	0	2.875344	2.497770	-2.058096
45	6	0	2.716966	1.833174	-3.302784
46	6	0	3.568603	2.162522	-4.369881
47	1	0	3.432212	1.645389	-5.320771
48	6	0	4.553789	3.143989	-4.269729
49	6	0	4.666971	3.828455	-3.054796
50	1	0	5.409041	4.621834	-2.956623
51	6	0	3.861564	3.525230	-1.954174
52	6	0	1.642023	0.809951	-3.594814
53	1	0	1.978609	-0.210002	-3.375320
54	1	0	1.373217	0.844953	-4.659035
55	1	0	0.739850	0.985564	-3.005685
56	6	0	5.471017	3.458074	-5.429212
57	1	0	6.445244	2.960908	-5.304693
58	1	0	5.662636	4.536963	-5.502957
59	1	0	5.041409	3.114785	-6.378968
60	6	0	4.065704	4.322212	-0.680988
61	1	0	3.146895	4.844530	-0.381636
62	1	0	4.858648	5.067648	-0.820111
63	1	0	4.339843	3.663326	0.154945
64	6	0	0.757853	3.454450	-0.170939
65	6	0	0.720578	3.980515	1.152524
66	6	0	-0.122036	5.058180	1.444185
67	1	0	-0.147106	5.438933	2.465722
68	6	0	-0.922763	5.660630	0.472759
69	6	0	-0.882424	5.133962	-0.817979
70	1	0	-1.518794	5.571493	-1.587469
71	6	0	-0.070221	4.046231	-1.164462
72	6	0	1.569455	3.424740	2.274576
73	1	0	1.411622	2.352278	2.429246
74	1	0	1.338766	3.939057	3.215901
75	1	0	2.639796	3.542758	2.063582
76	6	0	-1.787462	6.853807	0.798224
77	1	0	-1.276094	7.792516	0.534196
78	1	0	-2.023593	6.889629	1.868535
79	1	0	-2.730023	6.816044	0.238575
80	6	0	-0.162597	3.542912	-2.583815
81	1	0	0.813047	3.513531	-3.077889
82	1	0	-0.828185	4.188272	-3.170577
83	1	0	-0.586027	2.529946	-2.600253
84	15	0	-2.410902	1.163958	-0.854854
85	15	0	-1.615010	-2.314699	-0.577881
86	14	0	-0.965140	-0.285419	0.118658
87	6	0	-3.070978	2.527124	0.206252
88	6	0	-4.000241	3.380308	-0.455070
89	6	0	-4.586341	4.436387	0.248799
90	1	0	-5.308301	5.069189	-0.269316
91	6	0	-4.272523	4.706696	1.583597
92	6	0	-3.324597	3.891842	2.200020
93	1	0	-3.044597	4.095809	3.233776
94	6	0	-2.715975	2.811682	1.546477
95	6	0	-4.399894	3.192525	-1.904394
96	1	0	-5.002773	2.285332	-2.045854
97	1	0	-4.983880	4.053515	-2.251953
98	1	0	-3.515251	3.087247	-2.548593
99	6	0	-4.914233	5.862957	2.313530
100	1	0	-4.533448	6.823953	1.937635
101	1	0	-6.003858	5.865302	2.172177

102	1	0	-4.705719	5.817052	3.389875
103	6	0	-1.733104	1.991011	2.342833
104	1	0	-0.766808	1.905135	1.835967
105	1	0	-1.553703	2.450240	3.322292
106	1	0	-2.108574	0.971272	2.512344
107	6	0	-3.859814	0.068396	-1.239209
108	6	0	-3.878157	-0.513807	-2.534171
109	6	0	-4.911627	-1.392074	-2.876349
110	1	0	-4.909261	-1.842066	-3.869841
111	6	0	-5.936618	-1.715307	-1.982942
112	6	0	-5.916588	-1.117083	-0.722368
113	1	0	-6.698026	-1.363654	-0.004019
114	6	0	-4.900432	-0.238846	-0.327407
115	6	0	-2.797805	-0.237280	-3.556913
116	1	0	-1.823451	-0.598373	-3.201986
117	1	0	-3.027089	-0.743532	-4.502942
118	1	0	-2.693713	0.839675	-3.748007
119	6	0	-7.003460	-2.718927	-2.347566
120	1	0	-6.725455	-3.716612	-1.976036
121	1	0	-7.968844	-2.456735	-1.894784
122	1	0	-7.133924	-2.787693	-3.435170
123	6	0	-4.964783	0.323444	1.072455
124	1	0	-5.338794	1.355917	1.075960
125	1	0	-5.624332	-0.293202	1.694208
126	1	0	-3.974033	0.348641	1.532190
127	6	0	-3.033228	-3.078433	0.333306
128	6	0	-3.538753	-2.730211	1.610075
129	6	0	-4.769572	-3.262429	2.027614
130	1	0	-5.157155	-2.968001	3.004084
131	6	0	-5.502180	-4.157718	1.252462
132	6	0	-4.955010	-4.552594	0.026848
133	1	0	-5.488953	-5.278557	-0.587422
134	6	0	-3.754534	-4.026680	-0.451712
135	6	0	-2.824009	-1.875003	2.631764
136	1	0	-3.519325	-1.168890	3.101056
137	1	0	-2.411850	-2.513948	3.425785
138	1	0	-1.990479	-1.308117	2.212262
139	6	0	-6.848355	-4.674229	1.702660
140	1	0	-7.660765	-4.169787	1.157249
141	1	0	-6.946624	-5.750843	1.508408
142	1	0	-7.004214	-4.498110	2.774368
143	6	0	-3.265474	-4.488119	-1.809394
144	1	0	-2.244424	-4.890078	-1.757971
145	1	0	-3.924774	-5.270441	-2.205733
146	1	0	-3.252750	-3.649605	-2.519110
147	6	0	-0.153994	-3.448133	-0.532184
148	6	0	0.573724	-3.573490	-1.745820
149	6	0	1.701713	-4.401190	-1.786517
150	1	0	2.264397	-4.474578	-2.717433
151	6	0	2.133282	-5.123216	-0.671685
152	6	0	1.390349	-5.009332	0.506393
153	1	0	1.713337	-5.556712	1.392379
154	6	0	0.266176	-4.183705	0.603923
155	6	0	0.194623	-2.815739	-2.999684
156	1	0	0.236118	-1.730703	-2.827730
157	1	0	0.883174	-3.060448	-3.818108
158	1	0	-0.829418	-3.051869	-3.317514
159	6	0	3.386679	-5.961964	-0.717454
160	1	0	3.255228	-6.901912	-0.164434
161	1	0	3.668234	-6.199935	-1.751114
162	1	0	4.220934	-5.415093	-0.256785

163	6	0	-0.422777	-4.064022	1.938610
164	1	0	-1.451164	-4.443619	1.903004
165	1	0	0.129416	-4.618189	2.707479
166	1	0	-0.473192	-3.012838	2.239914

Final electronic energy (au): -4739.93014736

NIMAG = 0

7_{min}:

Center Number	Atomic Number	Atomic Type	Coordinates (Angstroms) X	Y	Z
1	15	0	2.117249	-1.581284	-1.407958
2	15	0	-2.106223	-1.970126	-0.108663
3	14	0	-1.024664	-0.065129	0.369576
4	6	0	3.541499	-2.374140	-0.516528
5	6	0	4.450604	-3.022507	-1.407719
6	6	0	5.630320	-3.578881	-0.910168
7	1	0	6.306598	-4.076567	-1.606457
8	6	0	5.972370	-3.505727	0.444864
9	6	0	5.077066	-2.873265	1.301976
10	1	0	5.316588	-2.807404	2.363834
11	6	0	3.862648	-2.321453	0.861124
12	6	0	4.187378	-3.142914	-2.897046
13	1	0	3.239554	-3.658552	-3.102406
14	1	0	4.996391	-3.704677	-3.380431
15	1	0	4.119314	-2.151893	-3.367666
16	6	0	7.267297	-4.100494	0.948548
17	1	0	7.365476	-3.975329	2.034127
18	1	0	8.134504	-3.623009	0.469537
19	1	0	7.323072	-5.174800	0.721126
20	6	0	2.970543	-1.775742	1.949773
21	1	0	2.205209	-1.089334	1.578598
22	1	0	3.571493	-1.244954	2.694759
23	1	0	2.453900	-2.602516	2.458108
24	6	0	0.878808	-2.899483	-1.801212
25	6	0	0.243658	-2.807646	-3.069768
26	6	0	-0.606842	-3.834813	-3.487299
27	1	0	-1.089847	-3.749561	-4.461318
28	6	0	-0.862539	-4.957121	-2.692088
29	6	0	-0.268722	-5.010717	-1.431151
30	1	0	-0.501292	-5.843184	-0.767514
31	6	0	0.582868	-4.002063	-0.962373
32	6	0	0.417169	-1.607363	-3.974333
33	1	0	0.034302	-0.698083	-3.491157
34	1	0	-0.128749	-1.751805	-4.914755
35	1	0	1.475411	-1.420452	-4.201744
36	6	0	-1.768222	-6.063219	-3.181381
37	1	0	-2.682877	-5.655618	-3.633781
38	1	0	-2.057522	-6.729314	-2.359346
39	1	0	-1.267411	-6.671271	-3.949842
40	6	0	1.093941	-4.113494	0.451389
41	1	0	2.154199	-4.395179	0.484892
42	1	0	0.508631	-4.852275	1.009821

43	1	0	1.000684	-3.151997	0.963680
44	6	0	-2.219998	-3.219733	1.261708
45	6	0	-1.569500	-3.173736	2.521006
46	6	0	-1.602604	-4.303028	3.352265
47	1	0	-1.096844	-4.251087	4.317282
48	6	0	-2.265278	-5.479186	2.999055
49	6	0	-2.928900	-5.500744	1.769766
50	1	0	-3.475082	-6.397239	1.472964
51	6	0	-2.917210	-4.408070	0.896038
52	6	0	-0.848510	-1.957356	3.050413
53	1	0	0.047304	-1.716884	2.463888
54	1	0	-0.536217	-2.121946	4.089606
55	1	0	-1.488237	-1.066031	3.028294
56	6	0	-2.254112	-6.690118	3.903812
57	1	0	-1.455301	-7.390619	3.615294
58	1	0	-3.204856	-7.236448	3.846644
59	1	0	-2.079981	-6.402420	4.948642
60	6	0	-3.670296	-4.543122	-0.410051
61	1	0	-4.537311	-3.870434	-0.447191
62	1	0	-4.022993	-5.573135	-0.542449
63	1	0	-3.027090	-4.284820	-1.261504
64	6	0	-3.835232	-1.335149	-0.386068
65	6	0	-4.232400	-1.185489	-1.740765
66	6	0	-5.504258	-0.680513	-2.030771
67	1	0	-5.793552	-0.559788	-3.075619
68	6	0	-6.406836	-0.321807	-1.026263
69	6	0	-6.015121	-0.508971	0.300584
70	1	0	-6.704756	-0.239793	1.100867
71	6	0	-4.753993	-1.011281	0.644742
72	6	0	-3.320640	-1.537952	-2.897075
73	1	0	-2.424992	-0.903298	-2.913837
74	1	0	-3.847989	-1.409411	-3.851284
75	1	0	-2.958140	-2.570462	-2.826469
76	6	0	-7.744748	0.289432	-1.364245
77	1	0	-8.123466	-0.083087	-2.325239
78	1	0	-7.651746	1.382068	-1.441205
79	1	0	-8.489436	0.073732	-0.587211
80	6	0	-4.436731	-1.186767	2.110571
81	1	0	-4.382549	-2.248835	2.381683
82	1	0	-5.206285	-0.705834	2.726679
83	1	0	-3.468932	-0.739515	2.360226
84	15	0	-2.117288	1.581345	1.407919
85	15	0	2.106238	1.970233	0.108678
86	14	0	1.024690	0.065255	-0.369634
87	6	0	-3.541663	2.374022	0.516532
88	6	0	-4.450903	3.022128	1.407769
89	6	0	-5.630738	3.578284	0.910263
90	1	0	-6.307142	4.075741	1.606593
91	6	0	-5.972747	3.505207	-0.444784
92	6	0	-5.077321	2.872983	-1.301940
93	1	0	-5.316849	2.807116	-2.363797
94	6	0	-3.862803	2.321363	-0.861123
95	6	0	-4.187714	3.142450	2.897108
96	1	0	-3.239964	3.658197	3.102529
97	1	0	-4.996807	3.704072	3.380522
98	1	0	-4.119534	2.151402	3.367650
99	6	0	-7.267685	4.099973	-0.948455
100	1	0	-7.366825	3.972981	-2.033737
101	1	0	-8.134827	3.623975	-0.467870
102	1	0	-7.322490	5.174738	-0.722931
103	6	0	-2.970610	1.775882	-1.949818

104	1	0	-2.205104	1.089630	-1.578701
105	1	0	-3.571471	1.244996	-2.694808
106	1	0	-2.454179	2.602799	-2.458136
107	6	0	-0.878913	2.899640	1.801093
108	6	0	-0.243717	2.807858	3.069624
109	6	0	0.606793	3.835048	3.487089
110	1	0	1.089836	3.749836	4.461091
111	6	0	0.862452	4.957326	2.691834
112	6	0	0.268595	5.010867	1.430909
113	1	0	0.501145	5.843304	0.767228
114	6	0	-0.583008	4.002198	0.962204
115	6	0	-0.417157	1.607603	3.974242
116	1	0	-0.034270	0.698322	3.491088
117	1	0	0.128787	1.752102	4.914639
118	1	0	-1.475383	1.420657	4.201694
119	6	0	1.768053	6.063513	3.181075
120	1	0	2.682174	5.655989	3.634609
121	1	0	2.058265	6.728897	2.358785
122	1	0	1.266734	6.672326	3.948607
123	6	0	-1.094132	4.113566	-0.451543
124	1	0	-2.154441	4.395051	-0.485013
125	1	0	-0.508980	4.852471	-1.009977
126	1	0	-1.000717	3.152097	-0.963864
127	6	0	2.220190	3.219810	-1.261706
128	6	0	1.569746	3.173851	-2.521005
129	6	0	1.603051	4.303128	-3.352324
130	1	0	1.097323	4.251235	-4.317358
131	6	0	2.265887	5.479178	-2.999159
132	6	0	2.929406	5.500724	-1.769784
133	1	0	3.475679	6.397161	-1.472963
134	6	0	2.917504	4.408113	-0.896022
135	6	0	0.848602	1.957565	-3.050418
136	1	0	-0.047224	1.717169	-2.463880
137	1	0	0.536309	2.122208	-4.089603
138	1	0	1.488236	1.066170	-3.028337
139	6	0	2.255261	6.690040	-3.904014
140	1	0	1.460470	7.393652	-3.612010
141	1	0	3.208319	7.232800	-3.851126
142	1	0	2.075495	6.402868	-4.948021
143	6	0	3.670470	4.543127	0.410139
144	1	0	4.537431	3.870375	0.447374
145	1	0	4.023237	5.573113	0.542553
146	1	0	3.027166	4.284888	1.261536
147	6	0	3.835214	1.335194	0.386186
148	6	0	4.232329	1.185558	1.740909
149	6	0	5.504151	0.680557	2.030982
150	1	0	5.793391	0.559844	3.075847
151	6	0	6.406756	0.321782	1.026512
152	6	0	6.015105	0.508923	-0.300348
153	1	0	6.704760	0.239681	-1.100593
154	6	0	4.753998	1.011271	-0.644576
155	6	0	3.320524	1.538079	2.897165
156	1	0	2.424824	0.903500	2.913874
157	1	0	3.847807	1.409505	3.851404
158	1	0	2.958114	2.570620	2.826532
159	6	0	7.744601	-0.289529	1.364622
160	1	0	8.124095	0.084322	2.324798
161	1	0	7.651147	-1.382000	1.443384
162	1	0	8.488905	-0.075391	0.586794
163	6	0	4.436820	1.186729	-2.110427
164	1	0	4.382794	2.248796	-2.381583

165	1	0	5.206338	0.705666	-2.726479
166	1	0	3.468974	0.739601	-2.360115

Final electronic energy (au): -4739.92527598

NIMAG = 0

7_{alt}:

Center Number	Atomic Number	Atomic Type	Coordinates (Angstroms) X	Y	Z
1	15	0	-0.512332	0.138927	1.699291
2	15	0	3.397755	0.737756	1.252588
3	14	0	1.678395	-0.751660	1.113572
4	6	0	-1.194661	1.688192	2.482424
5	6	0	-2.513261	1.674408	3.012122
6	6	0	-3.043328	2.863997	3.533078
7	1	0	-4.060175	2.845639	3.926094
8	6	0	-2.329999	4.062409	3.550173
9	6	0	-1.033837	4.053220	3.028142
10	1	0	-0.448999	4.973341	3.027193
11	6	0	-0.455509	2.897259	2.494815
12	6	0	-3.408634	0.456514	3.040726
13	1	0	-3.129500	-0.226552	3.852291
14	1	0	-4.450359	0.761100	3.185672
15	1	0	-3.360351	-0.109696	2.106670
16	6	0	-2.949126	5.335158	4.079437
17	1	0	-3.363629	5.938364	3.257298
18	1	0	-3.767408	5.118028	4.777584
19	1	0	-2.203260	5.953737	4.595658
20	6	0	0.942979	2.983054	1.951469
21	1	0	0.990908	2.569204	0.941922
22	1	0	1.295030	4.019802	1.917572
23	1	0	1.640925	2.404589	2.571357
24	6	0	-0.870356	-1.176269	2.978335
25	6	0	-1.695609	-2.298312	2.755178
26	6	0	-1.823802	-3.263624	3.766503
27	1	0	-2.460382	-4.129460	3.579318
28	6	0	-1.166697	-3.152533	4.989497
29	6	0	-0.351148	-2.031943	5.193743
30	1	0	0.180733	-1.923977	6.139867
31	6	0	-0.181561	-1.045541	4.219184
32	6	0	-2.486332	-2.527675	1.491953
33	1	0	-2.129292	-1.925383	0.655869
34	1	0	-2.437374	-3.582229	1.195299
35	1	0	-3.543445	-2.275118	1.645122
36	6	0	-1.311684	-4.206634	6.062973
37	1	0	-0.340583	-4.667443	6.294992
38	1	0	-1.693177	-3.768890	6.996571
39	1	0	-2.000687	-5.000137	5.748571
40	6	0	0.739967	0.113744	4.533066
41	1	0	0.196469	1.066980	4.553527
42	1	0	1.217023	-0.034843	5.509053
43	1	0	1.536782	0.214160	3.780727
44	6	0	3.932606	2.413051	0.634747

45	6	0	3.318068	3.162123	-0.391335
46	6	0	3.777176	4.456851	-0.676774
47	1	0	3.288530	5.019142	-1.474432
48	6	0	4.845755	5.034173	0.006709
49	6	0	5.466083	4.272298	1.004590
50	1	0	6.312521	4.694255	1.548554
51	6	0	5.030907	2.986899	1.340449
52	6	0	2.213475	2.626717	-1.264296
53	1	0	1.357956	3.315690	-1.276703
54	1	0	2.566306	2.517247	-2.298716
55	1	0	1.854051	1.650176	-0.933657
56	6	0	5.313439	6.438476	-0.300697
57	1	0	4.963073	7.147036	0.465367
58	1	0	6.410340	6.496327	-0.318962
59	1	0	4.931613	6.779955	-1.271175
60	6	0	5.764958	2.254559	2.445082
61	1	0	6.237584	1.334866	2.074670
62	1	0	6.542834	2.896344	2.876919
63	1	0	5.073160	1.951090	3.243566
64	6	0	4.756040	-0.430692	0.749076
65	6	0	5.183719	-1.405814	1.693773
66	6	0	6.169666	-2.332088	1.329821
67	1	0	6.481354	-3.077400	2.062744
68	6	0	6.773743	-2.320181	0.070371
69	6	0	6.377858	-1.328319	-0.830248
70	1	0	6.847505	-1.285154	-1.813999
71	6	0	5.385330	-0.388429	-0.524819
72	6	0	4.628176	-1.482565	3.101099
73	1	0	3.588552	-1.837537	3.102893
74	1	0	5.231730	-2.166306	3.711930
75	1	0	4.619990	-0.490448	3.571814
76	6	0	7.805784	-3.356130	-0.312770
77	1	0	8.348125	-3.721730	0.568943
78	1	0	7.330344	-4.226810	-0.790969
79	1	0	8.534410	-2.947305	-1.025144
80	6	0	5.038432	0.629142	-1.585281
81	1	0	5.410112	1.625939	-1.317770
82	1	0	5.477667	0.337477	-2.545034
83	1	0	3.955808	0.708960	-1.719888
84	1	0	-5.162090	6.361636	-3.403595
85	6	0	-5.282911	5.474419	-4.038512
86	6	0	-4.718362	4.242886	-3.368140
87	1	0	-4.768851	5.675532	-4.991109
88	1	0	-6.350574	5.349748	-4.263397
89	6	0	-3.738649	4.339309	-2.380296
90	6	0	-5.148301	2.962932	-3.726648
91	6	0	-3.181314	3.216837	-1.748014
92	1	0	-3.402872	5.328737	-2.066757
93	1	0	-5.920867	2.853656	-4.488936
94	6	0	-4.614773	1.807865	-3.145755
95	6	0	-3.610808	1.917955	-2.135448
96	6	0	-2.170784	3.502217	-0.659794
97	6	0	-5.153568	0.474429	-3.624413
98	15	0	-2.983175	0.279115	-1.528430
99	1	0	-1.140886	3.335350	-1.003862
100	1	0	-2.252249	4.546705	-0.332778
101	1	0	-2.305908	2.867410	0.221842
102	1	0	-5.705596	-0.047112	-2.831398
103	1	0	-5.827630	0.619866	-4.477669
104	1	0	-4.336523	-0.195266	-3.929054
105	14	0	-0.939904	0.515742	-0.513974

106	6	0	-4.394451	-0.466165	-0.583756
107	15	0	0.501408	-1.212170	-1.052595
108	6	0	-4.663140	-1.848034	-0.798288
109	6	0	-5.258135	0.271657	0.267970
110	6	0	1.654144	-0.873516	-2.466698
111	6	0	-0.082282	-2.968021	-1.389260
112	6	0	-5.745830	-2.452255	-0.153102
113	6	0	-3.801520	-2.706387	-1.692296
114	6	0	-6.332532	-0.382923	0.888916
115	6	0	-5.078376	1.740822	0.560897
116	6	0	2.801213	-1.714613	-2.560234
117	6	0	1.457989	0.138233	-3.445516
118	6	0	0.046957	-4.028265	-0.450264
119	6	0	-0.682357	-3.246988	-2.651709
120	1	0	-5.929904	-3.513252	-0.327632
121	6	0	-6.593754	-1.740577	0.701856
122	1	0	-2.795520	-2.832473	-1.278363
123	1	0	-4.239298	-3.705130	-1.811332
124	1	0	-3.676330	-2.251501	-2.682818
125	1	0	-6.981079	0.195220	1.548404
126	1	0	-5.387001	2.363136	-0.288036
127	1	0	-5.672782	2.029629	1.436150
128	1	0	-4.031983	1.979007	0.767829
129	6	0	3.700972	-1.531226	-3.614174
130	6	0	3.122085	-2.811674	-1.569271
131	6	0	2.418053	0.296132	-4.456590
132	6	0	0.257720	1.056735	-3.508931
133	6	0	-0.445504	-5.300125	-0.779698
134	6	0	0.729016	-3.902867	0.890013
135	6	0	-1.119632	-4.546000	-2.936607
136	6	0	-0.941525	-2.199843	-3.708050
137	6	0	-7.745228	-2.426969	1.400154
138	1	0	4.573003	-2.182558	-3.668176
139	6	0	3.541482	-0.522835	-4.564858
140	1	0	2.403252	-3.637403	-1.636956
141	1	0	4.128658	-3.201792	-1.752177
142	1	0	3.106623	-2.439025	-0.537573
143	1	0	2.261973	1.080220	-5.198022
144	1	0	0.279450	1.813225	-2.716884
145	1	0	0.236431	1.579755	-4.473148
146	1	0	-0.686795	0.514739	-3.403897
147	1	0	-0.346257	-6.097621	-0.043044
148	6	0	-1.027828	-5.587192	-2.013599
149	1	0	0.292869	-3.121538	1.516799
150	1	0	0.654142	-4.847149	1.442993
151	1	0	1.791116	-3.656719	0.774249
152	1	0	-1.574882	-4.735101	-3.909064
153	1	0	-0.014099	-1.833444	-4.161355
154	1	0	-1.572024	-2.615523	-4.503068
155	1	0	-1.473485	-1.345132	-3.272660
156	1	0	-8.377652	-2.967593	0.681704
157	1	0	-7.380903	-3.165474	2.129810
158	1	0	-8.372900	-1.703669	1.935448
159	6	0	4.575427	-0.299922	-5.642503
160	6	0	-1.567640	-6.962393	-2.328938
161	1	0	5.403190	0.317905	-5.260755
162	1	0	5.006117	-1.250612	-5.983016
163	1	0	4.145314	0.220345	-6.507616
164	1	0	-2.651710	-7.007663	-2.143340
165	1	0	-1.405586	-7.219572	-3.383920
166	1	0	-1.091241	-7.728897	-1.704714

––

Final electronic energy (au): -4739.89802246

NIMAG = 0

References:

[S1] R. A. Bartlett, M. M. Olmstead, P. P. Power, G. A. Sigel, *Inorg. Chem.* **1987**, *26*, 1941.

[S2] (a) K. Izod, D. G. Rayner, S. M. El-Hamruni, R. W. Harrington, U. Baisch, *Angew. Chem. Int. Ed.* **2014**, *53*, 3636. (b) K. Izod, P. Evans, P. G. Waddell, M. R. Probert, *Inorg. Chem.* **2016**, *55*, 10510.

[S3] CrysAlisPro, Agilent Technologies, Version 1.171.36.

[S4] R. C. Clark, J. S. Reid, *Acta Cryst.* **1995**, *A51*, 887.

[S5] G. M. Sheldrick, *Acta Cryst.* **2015**, *A71*, 3.

[S6] G. M. Sheldrick, *Acta Cryst.* **2008**, *A64*, 112.

[S7] O. V. Dolomanov, L. J. Bourhis, R. J. Gildea, J. A. K. Howard, H. Puschmann, *J. Appl. Cryst.*, **2009**, *42*, 339.

[S8] Gaussian 09, Revision E.01, M. J. Frisch, G. W. Trucks, H. B. Schlegel, G. E. Scuseria, M. A. Robb, J. R. Cheeseman, G. Scalmani, V. Barone, B. Mennucci, G. A. Petersson, H. Nakatsuji, M. Caricato, X. Li, H. P. Hratchian, A. F. Izmaylov, J. Bloino, G. Zheng, J. L. Sonnenberg, M. Hada, M. Ehara, K. Toyota, R. Fukuda, J. Hasegawa, M. Ishida, T. Nakajima, Y. Honda, O. Kitao, H. Nakai, T. Vreven, J. A. Montgomery, Jr., J. E. Peralta, F. Ogliaro, M. Bearpark, J. J. Heyd, E. Brothers, K. N. Kudin, V. N. Staroverov, R. Kobayashi, J. Normand, K. Raghavachari, A. Rendell, J. C. Burant, S. S. Iyengar, J. Tomasi, M. Cossi, N. Rega, J. M. Millam, M. Klene, J. E. Knox, J. B. Cross, V. Bakken, C. Adamo, J. Jaramillo, R. Gomperts, R. E. Stratmann, O. Yazyev, A. J. Austin, R. Cammi, C. Pomelli, J. W. Ochterski, R. L. Martin, K. Morokuma, V. G. Zakrzewski, G. A. Voth, P. Salvador, J. J. Dannenberg, S. Dapprich, A. D. Daniels, Ö. Farkas, J. B. Foresman, J. V. Ortiz, J. Cioslowski, D. J. Fox, Gaussian, Inc., Wallingford CT, **2009**.

[S9] S. Grimme, *J. Comput. Chem.* **2006**, *27*, 1787.

[S10] (a) A. D. McLean, G. S. Chandler, *J. Chem. Phys.* **1980**, *72* 5639; (b) K. Raghavachari, J. S. Binkley, R. Seeger, J. A. Pople, *J. Chem. Phys.*, **1980**, *72*, 650; (c) R. C. Binning Jr., L. A. Curtiss, *J. Comput. Chem.* **1990**, *11*, 1206; (d) M. P. McGrath, L. Radom, *J. Chem. Phys.*, **1991**, *94*, 511; (e) L. A. Curtiss, M. P. McGrath, J.-P. Blaudeau, N. E. Davis, R. C. Binning Jr., L. Radom, *J. Chem. Phys.* **1995**, *103*, 6104. (f) T. Clark, J. Chandrasekhar, G. W. Spitznagel, P. v. R. Schleyer, *J. Comput. Chem.* **1983**, *4*, 294.

[S11] (a) S. Simon, M. Duran, J. J. Dannenberg, *J. Chem. Phys.* **1996**, *105*, 11024. (b) S. F. Boys, F. Bernardi, *Mol. Phys.* **1970**, *19*, 553.

[S12] (a) R. McWeeny, *Phys. Rev.*, **1962**, *126*, 1028. (b) R. Ditchfield, *Mol. Phys.*, **1974**, *27*, 789. (c) K. Wolinski, J. F. Hilton, P. Pulay, *J. Am. Chem. Soc.*, **1990**, *112*, 8251. (d) J. R. Cheeseman, G. W. Trucks, T. A. Keith, M. J. Frisch, *J. Chem. Phys.*, **1996**, *104*, 5497.

[S13] NBO Version 3.1, E. D. Glendening, A. E. Reed, J. E. Carpenter, F. Weinhold.
